# Universal Architectural Concepts Underlying Protein Folding Patterns

**DOI:** 10.3389/fmolb.2020.612920

**Published:** 2021-04-30

**Authors:** Arun S. Konagurthu, Ramanan Subramanian, Lloyd Allison, David Abramson, Peter J. Stuckey, Maria Garcia de la Banda, Arthur M. Lesk

**Affiliations:** ^1^ Department of Data Science and Artificial Intelligence, Faculty of Information Technology, Monash University, Clayton, VIC, Australia; ^2^ Research Computing Center, University of Queensland, Brisbane, QLD, Australia; ^3^ School of Computing and Information Systems, University of Melbourne, Melbourne, VIC, Australia; ^4^ Department of Biochemistry and Molecular Biology, Pennsylvania State University, University Park, PA, United States; ^5^ MRC Laboratory of Molecular Biology, Cambridge, United Kingdom

**Keywords:** architectural concepts, protein-building blocks, structural motifs, lossless compression, information theory, folding pattern

## Abstract

What is the architectural “basis set” of the observed universe of protein structures? Using information-theoretic inference, we answer this question with a dictionary of 1,493 substructures—called *concepts*—typically at a subdomain level, based on an unbiased subset of known protein structures. Each *concept* represents a topologically conserved assembly of helices and strands that make contact. Any protein structure can be dissected into instances of concepts from this dictionary. We dissected the Protein Data Bank and completely inventoried all the concept instances. This yields many insights, including correlations between concepts and catalytic activities or binding sites, useful for rational drug design; local amino-acid sequence–structure correlations, useful for *ab initio* structure prediction methods; and information supporting the recognition and exploration of evolutionary relationships, useful for structural studies. An interactive site, Proçodic, at http://lcb.infotech.monash.edu.au/prosodic (click), provides access to and navigation of the entire dictionary of concepts and their usages, and all associated information. This report is part of a continuing programme with the goal of elucidating fundamental principles of protein architecture, in the spirit of the work of Cyrus Chothia.

## 1 Introduction

The polypeptide chains of amino acids (primary structure) contain, in most proteins, regions that fold into helices and strands of sheets (secondary structure), which in turn assemble to give proteins their intricate three-dimensional shapes and folding patterns (tertiary and quaternary structures). As of April 2021, experimental methods have already provided more than 167,000 entries in the Protein Data Bank (PDB) (Berman et al., 2003), containing the three-dimensional coordinates of proteins and protein–nucleic acid complexes from a wide range of species.

Unraveling protein architecture and discovering the relationship among these major levels of structural description provide the key to understanding how proteins function, how their 3D folding patterns form, and how they evolve (Lesk, 2016). Investigations of protein folding patterns have revealed recurrent themes (Pauling and Corey, 1951; Pauling et al., 1951; Levitt and Chothia, 1976; Lesk and Rose, 1981; Chothia and Lesk, 1986; Richards and Kundrot, 1988), which form the basis for widely used hierarchical classifications of protein structures (Murzin et al., 1995; Orengo et al., 1997; Andreeva et al., 2013; Schaeffer et al., 2016). Nevertheless, many aspects of the relationships across structural levels remain unresolved. Further, François Jacob observed that proteins evolve by “*bricolage*,” that is, through evolutionary tinkering by reusing “pieces” from other proteins (Jacob, 1977; Duboule and Wilkins, 1998). Despite much previous work to unravel these “pieces,” the problem of precisely characterizing them has remained open.

Chothia and Lesk (1986) introduced the idea of a *core* of the folding patterns of homologous proteins. This core comprises a maximal set of secondary structural elements (SSEs) that assemble in a common 3D topology, while withstanding a certain amount of distortion. The parts outside the core are structurally more variable.

Many related proteins share some but not all of the substructures that form their cores. Therefore, it is of great interest to discover the nature of the substructures that contribute to the cores of protein families. Some of these are *supersecondary structures*—small recurrent combinations of *successive* elements of secondary structure, such as the *β-α-β* subunit. Supersecondary structures recur within many protein folds and can be shared even by unrelated proteins. For example, the *β-α-β* subunit appears in NAD-binding domains, in TIM barrels, and in many other proteins.

Early definitions of supersecondary structures relied strongly on experts’ spotting and naming them (Rao and Rossmann, 1973; Kister, 2013). With the steady growth of the PDB, several methods have been developed to identify automatically, with varying operational definitions, a *library* of substructures that form what can be considered as the 3D building blocks of protein structures (Unger et al., 1989; Rooman et al., 1990; Unger and Sussman, 1993; Camproux et al., 1999; Micheletti et al., 2000; Kolodny et al., 2002; Friedberg and Godzik, 2005; Joseph et al., 2010; Chitturi et al., 2016; Dybas and Fiser, 2016; Mackenzie et al., 2016; Nepomnyachiy et al., 2017; de Oliveira et al., 2018; Joshi, 2018). However, these approaches have yielded limited libraries containing mostly short oligopeptide fragments, or assemblies of typically 2–4 secondary structural elements. It has been a challenge so far to go further than that and dissect protein structures into a more complete set that includes *larger* conserved substructures. (A more detailed exploration of key prior work on this topic is provided under “Comparison with previous work” within the “Results” section.) Apart from the enormous computational challenge this problem poses, the attempts made so far have lacked a rigorous framework in which to describe, compute, identify, and resolve a dictionary of conserved assemblies of secondary structures.

Thus, the key focus of this work is to go beyond definitions of recurring substructural patterns that are identified using *ad hoc* formulations and adjustments. This work utilizes new statistical models to describe all observed protein folding patterns in terms of their substructural constituents. It provides an attempt toward a systematic description of recurrent substructures of protein folding patterns using methodological devices never previously explored in the literature on this topic. Finally, this work is broadly analogous (in scope and application) to finding a formalized description of “syntactic structures” that now underpins linguistic analyses of natural languages (Chomsky, 1957).

Specifically, this work unravels observed protein folding patterns into a dictionary of architectural building blocks (*concepts*) containing topologically conserved assemblies of helices and strands that make contact. We note that several databases such as SCOP (Murzin et al., 1995; Andreeva et al., 2013; Chandonia et al., 2017), CATH (Orengo et al., 1997), and ECOD (Schaeffer et al., 2016) classify protein structures at the level of domains, and include multiple instances of domains with very similar structures. *Concepts*, in contrast, provide a dictionary of independent structural patterns, into which full domains can be dissected.

We distinguish concepts both from motifs and from domains as follows:

• We understand the term motifs to mean recurrent structural patterns in proteins that can—in their entirety or partially—be superposed with low root-mean-square deviation of the backbone (or at least of the $C_{\alpha }$) atoms. The idea of a concept focuses instead on conservation of the *topology* of secondary structure assembly, but instances of the same concept in different proteins can less-rigidly preserve structure and have varying lengths.
• Domains in proteins are individual compact units. Although some concepts do correspond to domains, some are not in themselves entirely compact, some are subdomains, and others comprise portions or even all of multiple domains.

The determination of the dictionary was completely automatic (i.e., *unsupervised*), and unbiased by any previously known sequence or structural patterns. Our framework to infer this dictionary can be best understood as an imaginary communication between a transmitter and receiver pair over a communication (Shannon) channel. The transmitter has a collection of protein shapes she wants to share with the receiver. The transmitter has two possible methods of communication. The first involves communicating the collection *as is*—this constitutes the null or baseline model. But another approach is to communicate the whole collection more efficiently using a dictionary of concepts, followed by the details of the collection specified with the aid of that stated dictionary. Here, the role of the dictionary is to illuminate common patterns observed in the collection and is stated one-off over all shapes in the collection. It is intuitive to observe that the better a dictionary, in terms of its ability to describe (i.e., fit) the shapes in the collection, the more economical will be the description of the source collection. An optimal dictionary in this framework is the one that yields the most economical one-off statement of the dictionary and the collection using that dictionary.

Our approach relies on an information-theoretic framework that allows the inference of a dictionary that a) avoids overfitting (i.e., avoiding inferring a dictionary that is more complex than necessary to explain the observed folding patterns) and b) achieves an objective trade-off between the descriptive complexity of concepts in the dictionary and their fidelity (i.e., the amount of compression) gained when explaining the observed protein folding patterns. This dictionary of concepts advances the current knowledge of conserved subdomain structural patterns significantly beyond the classical supersecondary structures and other known patterns. Thus, this work presents a “basis set” of substructural concepts underlying all observed protein folding patterns, and allows any protein chain to be decomposed optimally into parts corresponding to substructures from this set. It thereby contributes a plethora of useful biological insights, such as the following:

1. Understanding the fundamental components of protein folding patterns. Our dictionary of concepts will support innovative projects aimed at the analysis of protein structures.
2. Correlation, in many cases, of concepts with functions directly, or indirectly *via* ligand-binding sites. This provides useful predictions in the case of proteins with known structure but unknown function.
3. Many concepts show amino-acid sequence correlation; that is, some conservation of sequence patterns. These results are applicable to protein structure prediction by suggesting conformations of local regions.

The results of dissecting all the structures in the current PDB, or of dissecting a user-supplied set of protein coordinates, are accessible from the Proçodic website: http://lcb.infotech.monash.edu.au/prosodic (click). This site supports the interactive exploration of protein structures and their relationships.

## 2 Results

### 2.1 Automatic Inference of a Dictionary of Substructural Concepts

**FIGURE 1:**
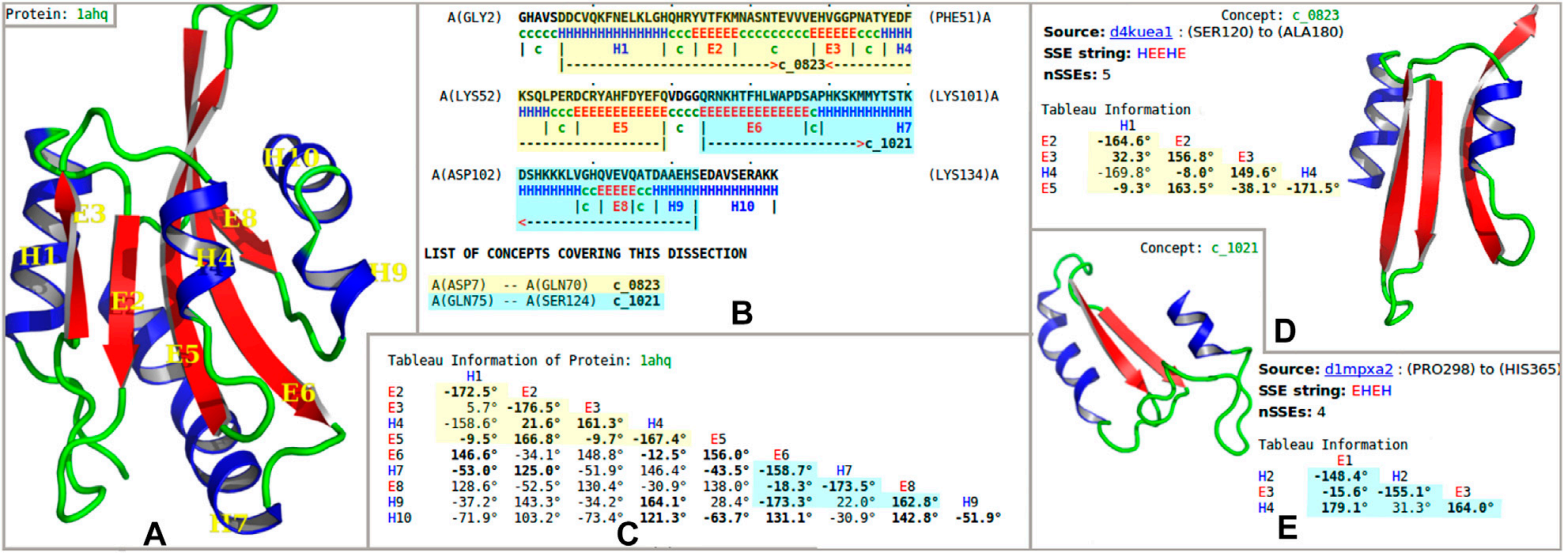
Example dissection of an actin-binding protein into “concepts” from the inferred dictionary. **(A)** Secondary-structural cartoon representation of the crystal structure of the actin-binding protein actophorin from *Acanthamoeba* (1ahq) (Leonard et al., 1997). **(B)** Secondary structural assignment [using SST (Konagurthu et al., 2012); H = helix, E = strand of sheet] and the optimal dissection of the protein chain into nonoverlapping regions, using the inferred concept dictionary. This information is shown with reference to the amino-acid sequence information in a marked-up format: the dissection of 1ahq uses *concepts* (see the text) c_0823 (highlighted in yellow) and c_1021 (highlighted in blue). **(C)** Tableau representation of the folding pattern of 1ahq. The highlighted subtableaux corresponds to concepts c_0823 and c_1021. Here, only the lower-triangle part of the tableau information is shown because the full tableau is a symmetric matrix. The rows and columns are indexed by secondary structure elements in order of appearance in the polypeptide chain. Off-diagonal elements record the angles between the pairs of secondary structural elements; boldface indicates that there is a contact between the corresponding pair of secondary structural elements. **(D**, **E)** The concepts c_0823 and c_1021 are shown, together with their *archetypal* tableaux and corresponding secondary structural representation.

This work uses the concise *tableau* representation of protein folding patterns introduced by Lesk (1995), which is based on the idea that the essence of a protein folding topology is captured by the order, patterns of contacts, and geometry of the assembly of secondary structural elements along the amino-acid chain. A tableau corresponds to the 3D structure of a single-protein domain (or sometimes chain), and has the form of a symmetric matrix (Figures 1A,C). Importantly, in this representation supersecondary structures can be defined in a compact and computable way as subtableaux containing two or more *successive* secondary structure elements in contact (Figures 1D,E).

We have constructed the dictionary reported here using our recently developed method to infer, automatically, conserved assemblies of secondary structural elements within *any* given source collection of tableaux (Subramanian et al., 2017). We call these substructures *concepts*. This idea of a concept is constrained by the requirement that every secondary structural element in the concept must be in contact with at least one other secondary-structure element in that concept. Our concept inference approach (Subramanian et al., 2017) is based on the minimum message length criterion for statistical inference (Wallace and Boulton, 1968; Wallace, 2005; Allison, 2018) and lossless data compression. We have applied this method to compress the source collection of tableaux corresponding to ASTRAL SCOP domains (Murzin et al., 1995; Andreeva et al., 2013; Chandonia et al., 2017). This has allowed us to infer a dictionary of 1,493 substructural concepts that *most concisely* and *losslessly* describes the entire source collection, and does so without any prior knowledge or preconceived notions regarding these recurrent substructures.

The total computational effort required to identify this dictionary is equivalent to about 7 years of runtime on a modern computer. Therefore, we parallelized our method and ran it on a high-performance computing cluster using 240 cores to identify the Proçodic dictionary in 14 days (see Section 4).

### 2.2 Proçodic: The Dictionary of Inferred Concepts

Each of the 1,493 concepts in the dictionary is designated by an identifier of the form “c_” followed by 4 digits: c_0001—c_1493. This order follows 1) the decreasing length in the number of secondary structural elements (nSSEs) defining each concept, and 2) for concepts containing the same number of SSEs, the lexicographic order of their secondary structural strings, where we represent any helix by “H” and any strand by “E.”

**FIGURE 2:**
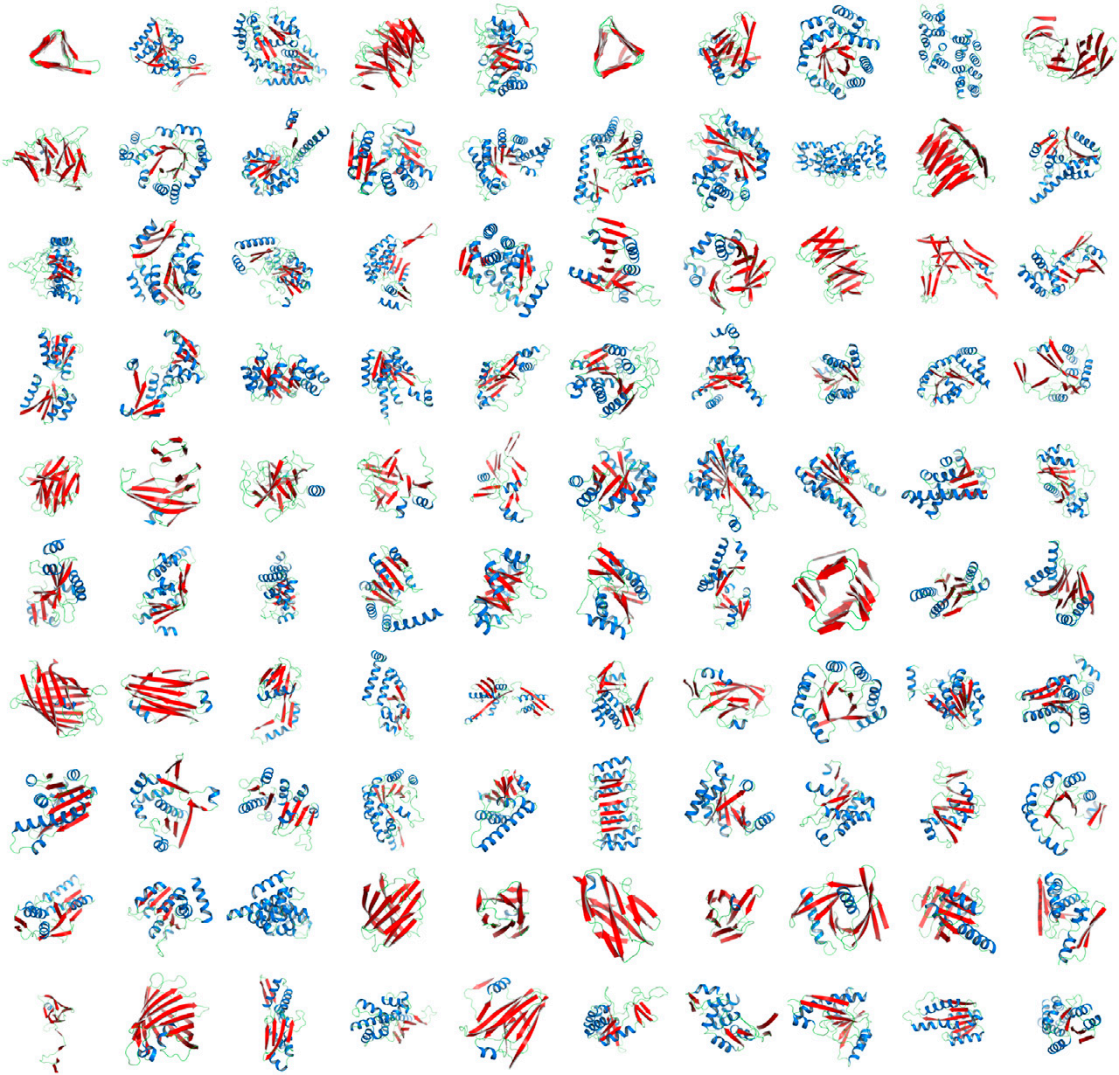
Top 100 concepts from the inferred dictionary. The representative structural cartoons of the top 100 concepts from the inferred dictionary containing 1,493 concepts, ranked in a decreasing order of number of secondary-structure elements (row-wise top-left to bottom-right: c_0001 to c_0100). Strands of sheet are shown in Red; helices in Blue. (See the website for the full interactive listing.) The inference of the whole dictionary was automatic without any prior knowledge or preconceived notions of these recurrent themes. The inferred concepts subsume known patterns; for example, shown in the figure are: “α-β Barrel” (c_0005), “Armadillo repeat” (c_0083), “β Barrel” (c_0061), “β Propeller” (c_0004), “Icosahedral virus coat protein” (c_0067), Immunoglobulin (c_0062), “Jellyroll architecture” (c_0084), “Left-handed β-Helix” (c_0001), “Leucine-rich repeat” (c_0076), “Right-handed quadrilateral β-Helix” (c_0058) “NAD-binding domain” (c_0002), “TIM barrel” (c_0008), etc. Other classical supersecondary structures not shown in this figure such as β-hairpin (c_1442), α-hairpin (c_1484), β-α-β unit (c_1240) appear lower down in the dictionary of concepts, ordered from largest to smallest.

**FIGURE 3:**
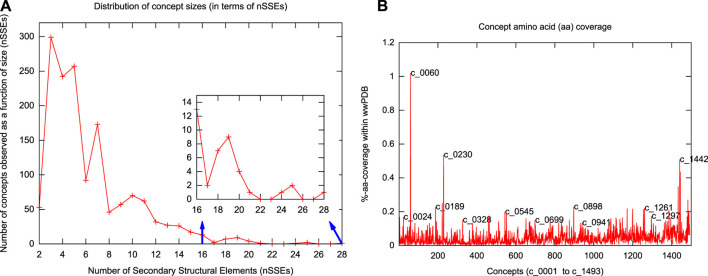
Distributions of concept length and amino-acid coverage. **(A)** Distribution of concept lengths in terms of the number of secondary structural elements (nSSEs) they contain. The smallest concepts have 2 secondary structural elements; the largest has 28. **(B)** Individual concept amino-acid coverage (as a percentage of the total 74,246,836 residues) in the serial order of concept identifiers (with some concepts highlighted).

Figure 2 shows the top 100 concepts in the dictionary, ordered by number of SSEs included. The largest concept (c_0001) contains 28 secondary structural elements. The smallest concepts (c_1441—c_1493)—not shown in Figure 2—contain only two elements. (Note that a single-helix or a single-strand/extended region is not considered here as a concept.) The distribution of inferred concept sizes is shown in Figure 3A: 9 concepts (c_0001—c_0009) are composed of an assembly of ≥20 secondary structural elements, 48 concepts (c_0010—c_0057) have between 15 and 19 SSEs, 217 concepts (c_0058—c_0274) contain between 10 and 14 SSEs, 217 concepts (c_0058—c_0274) contain between 10 and 14 SSEs, and 368 concepts (c_0275—c_0642) contain between 9 and 6 SSEs. The remaining concepts contain between 5 and 2 SSEs. The median concept size is 5 SSEs.

On average, a concept archetype is significantly smaller (with 47.6% of the number of SSEs) than its source protein domain. Yet, there are several concepts inferred in our dictionary that describe conserved folding patterns at the level of domains. These include: NAD-binding domain (c_0173), β-grasp fold (e.g., c_729), β-propeller (c_0382), Swiss/Jelly roll fold (c_0406), Ferredoxin (plait) fold (c_0581), TIM barrel (c_0008), Immunoglobulin fold (c_0118, c_0121), Ubiquitin roll (c_0737), and large β-barrel (c_0061). This shows that our dictionary encompasses a broader set of substructural invariants than previous studies (see Section 2.5). This advantage is due to our use of tableaux to capture concisely the essence of protein folding patterns, together with the information-theoretic criterion of minimum message length to yield an objective dictionary complexity-versus-fidelity trade-off.

The null model encoding length of our source collection is 33,352,380 bits. The encoding length after compressing the same collection using the inferred dictionary is 31,927,340 bits. The resultant compression is 1,425,040 bits (or 4.3%) over the null model. We emphasize that this compression gain is over the null model encodings of the tableaux representations which are themselves compact 2D representations of 3D structural information.

The complete inferred dictionary is available *via* the interactive website Proçodic (for Protein Concept dictionary—the cedilla allows the pronunciation as “prosodic”) at http://lcb.infotech.monash.edu.au/prosodic. As discussed later, this site allows the exploration of any structure that the user provides as input, or of specific concepts that are of motivating focus for the user, including: the usages of concepts in other structures, both homologous and nonhomologous; or the inspection of frequently occurring keywords within the “KEYWDS” records and the ligand-binding information from the “HETATM” records extracted from the source PDB coordinate files (see Section 3).

### 2.3 Our Dictionary Subsumes Known Supersecondary Structural Motifs

Our dictionary includes many concepts that match the known repertoire of supersecondary structural motifs (Efimov, 2013). Matched motifs involving assemblies of a small number of helices and strands include: antiparallel (c_1442) and parallel (c_1443) *β-β* assemblies, *α-α* hairpin (c_1484) *α-β/β-α* assembly, (c_1459/c_1472), basic helix–loop–helix (c_1351), *β-α-β* motif (c_1240), EF-Hand (c_1342, c_1491), *ϕ*-motif (c_1178), helix–turn–helix motifs (c_0826 – winged type I, c_0870 – winged type II, c_1373 – plain), four-helix bundle (c_1101 – type I, c_1117 – type II), *β*-meander (c_1187), Greek key (c_0964), Zinc finger (c_1230), helix–hairpin–helix motif (c_1068), *β*-sandwich (c_0390), and αβ-sandwich (c_0603), among others.

Our dictionary also includes larger assemblies of helices and strands that match known *repeating* structural motifs. These include three-sided left-handed *β*-helix (c_0001, c_0380), three-sided right-handed *β*-helix (c_0388), right-handed quadrilateral *β*-helix (c_0058), ankyrin repeat (c_0370, c_0632), armadillo repeat (c_0083, c_0888), kelch repeat (c_0395), *α*-solenoid (c_0270, c_0271), and leucine-rich repeat (c_0076), among others.

Proçodic yields a flat (nonhierarchical) dictionary of 1,493 concepts. The inference of these concepts is unsupervised, driven by information-theoretic trade-off between the dictionary complexity and its fidelity to explain the source collection of tableaux. Visual inspection reveals shared topological relationships between certain subsets of concepts (e.g., c_0001 and c_0006; see Figure 2). Therefore, to explore the topological relationships between the inferred concepts, we undertake an agglomerative clustering exercise to construct a hierarchy from that otherwise flat dictionary of concepts. We emphasize that this exercise is *not* meant to suggest any structural pathways [cf. Efimov structural trees (Efimov, 2013)] or evolutionary relationships between concepts, but merely provides a device to explore their topological relationships. (We also emphasize that a systematic approach to finding hierarchical relationships and structural pathways requires the unsupervised Bayesian inference of a hierarchical dictionary of concepts, which is beyond the scope of the current work.)

To undertake this agglomerative clustering, since each concept archetype defines a (sub)tableau derived from a tableau of the domain in the source collection, we can infer the dictionary of *meta-concepts* (i.e., concepts of “concepts”) that best explains all the Proçodic concept tableaux. This is achieved by using exactly the same unsupervised (flat dictionary) inference methodology that was used to infer Proçodic concepts. That is, we now treat the tableaux representing 1,493 archetypes from our inferred Proçodic concept dictionary as the source collection, and rerun our inference method (see Section 4). This in turn yielded 34 meta-concepts that dissect (i.e., best explain) the inferred 1,493 concepts. The text file containing these meta-concepts, along with the corresponding list of Proçodic concepts that use each meta-concept within their dissections, is available in the supporting data file: metaConceptsAndUsageList.txt (click).

This permits the decomposition of each Proçodic concept in terms of these 34 meta-concepts. Thus, each Proçodic concept can be represented as a 34-dimensional feature vector in the meta-concept space, where each vector component denotes the number of times the corresponding meta-concept is used in that concept dissection. We note that this representation is similar to the bag-of-words model (Harris, 1954) used in information retrieval and natural language processing. Using this feature vector representation, the 1,493 Proçodic concepts are clustered hierarchically using the following method:

1. A 1,493×1,493 similarity matrix between Proçodic concepts is constructed using the cosine similarity measure (Singhal, 2001) between all the pairs of these 34-dimensional vectors.
2. Using the resultant similarity matrix, we cluster all the Proçodic concepts hierarchically, based on the unweighted pair-groups method using arithmetic averages (Sokal, 1958).

This procedure yields a hierarchical tree of concept relationships, available in an interactive format from: prosodicConceptClustering.html (click). This tree reveals similarities that are also detectable by comparing the concept archetypes, their usages, and keywords. For example, c_0009 and c_0018 are both helical bundles related to the architecture of Annexin proteins, with c_0009 having one extra helix compared to c_0018. Another example is the cluster containing c_0001, c_0006, c_0113, and c_0380, where all represent left-handed *β*-helical motifs composed of 28, 20, 12, and 7 *β*-strands, respectively.

### 2.4 Dissection of PDB and Coverage of Concepts Across the Protein Folding Space

The methods used for this work also permit the optimal *dissection*, within seconds (on a single processor), of any protein chain into nonoverlapping regions that are explained (compressed) using the concepts from the inferred dictionary. Figure 1 shows an example of the dissection of the crystal structure of the actin-binding protein actophorin from *Acanthamoeba* (1ahq) (see the Proçodic website to dissect any protein structure of interest; either a PDB entry or a user-supplied coordinate set). We note that regions not assigned to any dictionary concept (notionally designated to the *null* concept, c_0000) remain uncompressed. These include the small set of proteins that have no secondary structure, for instance wheat-germ agglutinin (9wga).

We have dissected the entire PDB, which at the time of calculation resulted in tableaux corresponding to 275,014 protein chains containing 74,246,839 amino-acid residues overall. (Note that the dictionary was constructed using an *unbiased* set of domains from ASTRAL, but the subsequent dissection of the entire PDB reflects the biases in the distribution of protein folding patterns in the full database.) The usages of the resulting concepts cover regions within proteins that account for 66.35% (49,262,577) of the total (74,246,839) amino acids in the PDB protein chains we dissected (Supplementary Figure S3A). The remaining 33.65% is dominated by single secondary structural elements, plus loops between successive concept assignments along a dissected chain. Figure 3B shows the distributions of amino-acid coverage of concept usages within the PDB. Concept c_0060 has the largest coverage in terms of the number of amino acids its usages cover. This concept is composed of 14 secondary structural elements (SSE string: EEEEHHEEEEHHEE) assembling into a four-layer architecture, with its core containing two layers of closely packed five-stranded *β*-sheets (Chothia et al., 1977) that are sandwiched between two outer layers, containing two *α*-helices each (see Figure 2, the rightmost structure on the sixth row). In total, this concept was used within 3,892 protein chains, with a median value of amino-acid coverage equal to 194 residues (Supplementary Figures S3A,B). Examination of these usages reveals that they all come from the protein chains of 285 proteasome complexes. At the other extreme is concept c_0568, which has the smallest amino-acid coverage: 561 residues over 13 protein chains related to plant and bacterial Ferredoxins (Tagawa and Arnon, 1962). This concept is composed of 6 secondary structural elements (SSE string: EEHEEE).

**FIGURE 4:**
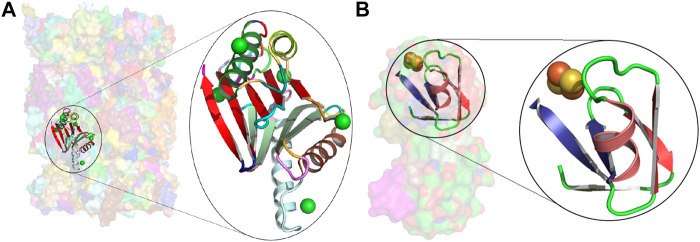
Usages of concepts c_0060 and c_0568. **(A)** Transparent surface rendering of the native human 20S proteasome at 1.8 Å (5le5), with the usage of concept c_0060 in the β5 subunit (chain Y in the amino-acid region THR1 to ASN191) shown in cartoon. The closeup of this region reveals a chloride ion in all active sites. Chloride ions are known to facilitate a proton shuttle catalytic mechanism (Schrader et al., 2016). **(B)** Similar rendering as above for the usage of concept c_0568 in the 2.3 Å Ferredoxin structure from *Mastigocladus laminosus* (3p63 chain A in the amino acid region THR48 to GLU90). The closeup shows the region linked to the $Fe_{2}S_{2}$-cluster binding.

Insights about the concepts can be gained from their usage information. For example, consider the concepts c_0060 and c_0568 mentioned earlier: the concept c_0060 covers the β5 subunit of a recently solved structure of the native human 20S proteasome at 1.8 Å resolution (5le5) (Schrader et al., 2016). This landmark study revealed a number of functionally important differences with respect to what was known from the previously published 20S proteasome structures. In particular, it identified chloride ions within all active sites, thus significantly revising the description of the proteasome active site, and providing new insights into peptide hydrolysis that underpin the “development of next-generation proteasome-based cancer therapeutics” (Schrader et al., 2016). The examination of the usages of c_0060 within the dissection of 5le5 (chain Y – β5 subunit) reveals that this concept is directly linked to proteolytic active sites (Figure 4A). Analyses of the human-annotated keywords used in the PDB coordinate files from these usages showed among its top 10 frequently used phrases terms such as “Cancer (therapy),” “Drug resistance,” and “Bortezomib”—an anticancer drug and the first therapeutic proteasome inhibitor to be used in humans. This is strong evidence of the concept’s link to a proteolytic active site. A similar examination of the usage instances of the concept c_0568 directly links it to the $Fe_{2}S_{2}$-cluster binding ferredoxins (see Figure 4B), which mediate electron transfer (Nechushtai et al., 2011).

**FIGURE 5:**
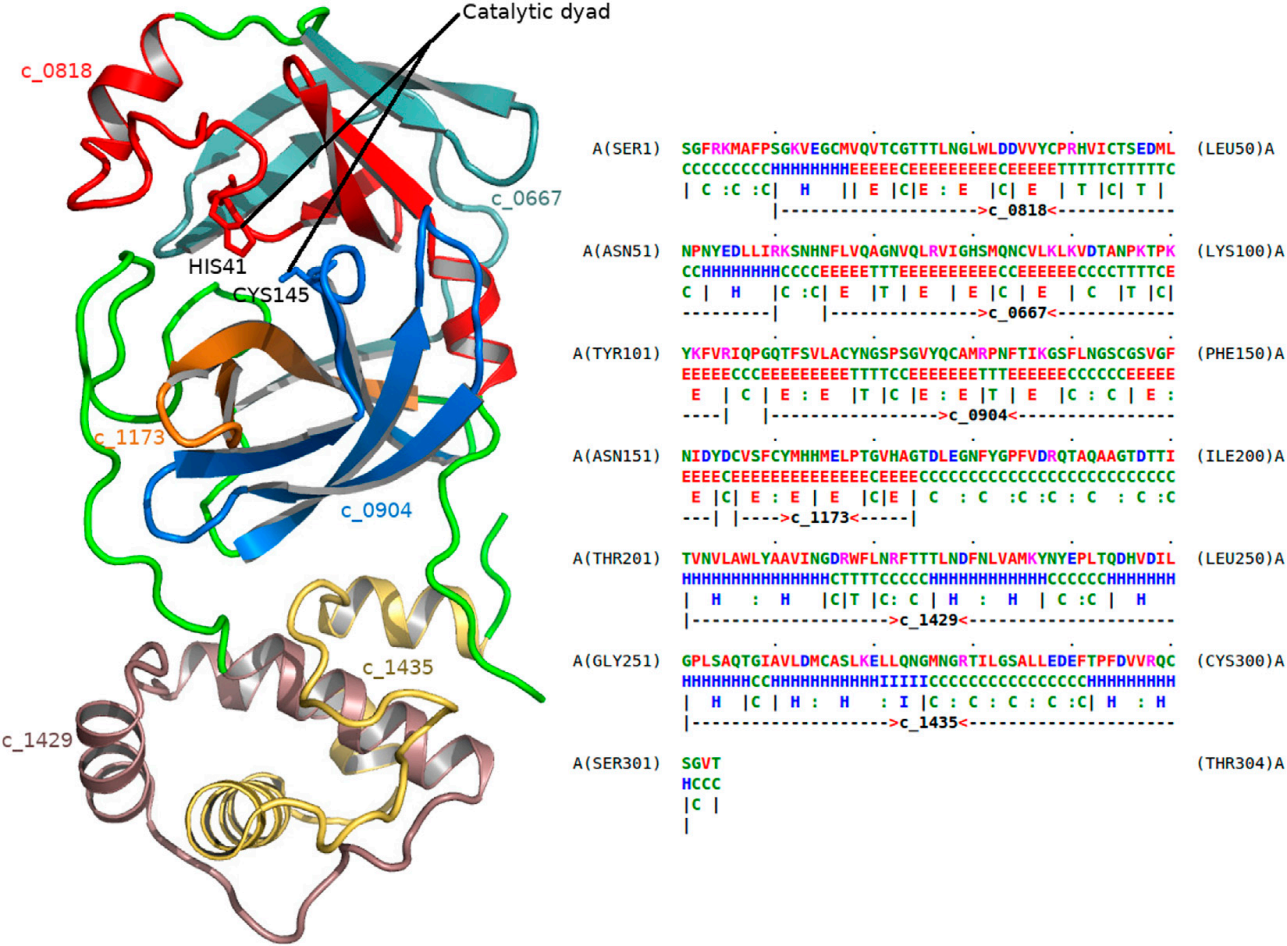
Dissection of the main protease of SARS-CoV-2 virus. The left frame shows the 1.8 Å crystal structure of the main protease of SARS-CoV-2 (5r84). The right frame gives the dissection of this protein as markup under 5r84’s amino-acid sequence (chain A). The successive regions of 5r84 chain A are explained using the following concepts (in that order): c_0818, c_0667, c_0904, c_1173, c_1429, and c_1435, respectively. Their corresponding substructural regions of the protease are shown with varying colors (left frame). Cysteine 145 (CYS145) and Histidine 41 (HIS41) residues form the catalytic dyad of this protease, and are associated with concepts c_0904 and c_0818, respectively.

As another example, consider the dissection of the main protease 5r84 (Figure 5) of the SARS-CoV-2 virus. This virus is the cause of the coronavirus disease (COVID-19). 5r84 is a cysteine protease that is responsible for cleaving the SARS-CoV-2 polyprotein chain that prepares the molecular machinery responsible for viral replication and infection. The dissection involves, among others, the following two concepts: c_0818 and c_0904. (For a full list of concepts in the dissection, see Figure 5.) Studying the usages of these concepts, it becomes clear that they are composed of highly conserved substructures that are specific to viral proteases, mainly coronaviruses (SARS and MERS). Concept c_0904 explains the region of 5r84 containing the catalytic cysteine-145 residue (CYS145) of this main protease, whereas c_0814 explains the other residue in the catalytic-dyad, histidine-41 (HIS41). Therefore, these concepts are directly linked to the catalytic function of the protease.

### 2.5 Comparison With Previous Work

Many previous studies have attempted to identify a canonical set of recurrent patterns that encompass the structures of proteins.

Dissection of protein folding patterns into substructures began with the recognition of recurrent patterns. The first of these were the canonical secondary structures (*α*-helix and *β*-sheet) followed by descriptions of supersecondary structures (*α*-hairpin, *β*-hairpin, and *β-α-β* unit). At this point the approach was observation and intuition-based rather than systematic, and the field lacked attempts to determine a set of substructures from which *complete* domain structures could be assembled. The earliest attempts to generate a roster of supersecondary structures automatically, with varying motivations, include those of Lesk and Rose (1981), Jones and Thirup (1986), and Richards and Kundrot (1988).

To identify a set of building blocks that *cover* protein structures, Unger et al. (1989) analyzed protein main chain conformations in terms of hexamers (oligopeptides of six amino-acid residues). Their analysis involved a refined set of 82 proteins in the (then) known structures, which contributed to a total of 12,973 hexamers. Using a normalized root-mean-square-deviation (RMSD)–based membership function (with an RMSD threshold of 1 Å) and a variant of *K*-nearest-neighbor clustering, they demonstrated that most hexamers grouped into 55 disjoint clusters.

Much subsequent work followed along similar lines of clustering short oligopeptide fragments using variations of clustering heuristics and membership-deciding thresholds to produce different local fragment libraries (Tramontano et al. 1989; Rooman et al., 1990; Hutchinson and Thornton, 1996; Micheletti et al., 2000; Kolodny et al., 2002; Kihara and Skolnick, 2003; Friedberg and Godzik, 2005; Joseph et al., 2010). For instance, Micheletti et al. (2000) sought a minimal set of “oligons” that can represent protein structures, by clustering oligopeptide conformations extracted from known structures. They considered oligopeptide lengths from 4 to 7 and created libraries containing 8, 202, 932, and 2561 elements—within which they recognized redundancies. They were able to fit a set of test structures to within an RMSD of approximately 1 Å.

The main limitations of these approaches are at least two-fold: 1) The nature of the covering substructures is *imposed*—in these cases, short oligopeptide fragments—rather than allowing their method to identify more general possibilities, and 2) the definition of cluster membership of various oligopeptide fragments remains extremely sensitive to the chosen RMSD threshold values and clustering heuristic.

Complementing the above strategies that rely on clustering local 3D fragments, Bystroff et al. (1996) and Bystroff and Baker (1998) proposed a fully automated method to cluster short 1D sequence segments into a library (I-sites) of amino-acid patterns that correlate strongly with their 3D (local) structure. These sequence segments were clustered using a weighted amino-acid frequency profile (Vingron and Argos, 1989) over a *K*-means clustering approach. Subsequently, over an iterative procedure, pairs of peptide segments within each cluster are evaluated based on their structural characteristics (C_*α*_–C_*α*_ distance profiles and backbone torsion angles) to select a “paradigm” local structure for their sequence cluster. Latest I-sites library (v5.3) reports 128 clusters containing motifs of length ranging from 3 to 15 amino acids. This popular library, together with the inferred local sequence–structure relationships, now underpins successful and popular *ab initio* structure prediction methods (Rohl et al., 2004). Despite being a noteworthy milestone in the literature, this library is not geared toward identifying topologically conserved assemblies of SSEs, which is the main focus of the work presented here.

Camproux et al. (1999) used an *a priori* method based on hidden Markov models (HMM) to identify a recurrent 3D structural alphabet. In their work, proteins are described using a sequence of overlapping tetrapeptide states on which a HMM is used to infer libraries of fragments together with their local conformational dependencies. This work mainly yielded 12 distinct tetrapeptide states derived from a data set of about 100 proteins. These states correspond predominantly to conformations of classical helices, strands, and turns, plus a few others. Further extension of this work (Camproux et al., 2004) gathered 27 tetrapeptide states. This work also examined the restrictions on the sequences of such states that appear in proteins. The inferred 27 tetrapeptide states correspond to α, $3_{10}$, and π helices, extended strands, turns of various descriptions and coil, respectively. Using different models, Pandini et al. (2010) also clustered tetrapeptide fragments (using the internal angles between the C_*α*_ coordinates) from known proteins to determine another structural alphabet. Nevertheless, similar to the other libraries, these structural alphabets remain extremely short and limited in scope.

Going beyond the clustering of oligopeptide fragments, some key studies have iteratively assembled SSEs under specific rules to explore structural “pathways” of observed protein folds. Specifically, Efimov (1997) used a constructive approach to introduce the notion of “structural trees.” These trees reveal how folding patterns can be constructed from root structural motifs *via* addition of helices and strands in a stepwise fashion, subject to a restricted set of growth-rules. Efimov examined five types of structural trees corresponding to five protein superfamilies. The key outcome of this work was the demonstration that the structural trees give pathways of growth that lead to known protein folding patterns. Murzin and Finkelstein (1988) presented a model for the possible arrangements of α-helices in globular proteins. Subsequently, Taylor (2002) also explored a similar idea. Taylor’s work constructed idealized topologies of protein structures by applying SSE packing rules that build on a set of basic “forms.” These forms are represented using stick models of SSEs in different layered arrangements, where the spacing between idealized helices (of arbitrary lengths) within a layer is fixed to 10 Å, whereas that between idealized strands is set to 5 Å. To match any protein to the sets of idealized forms, a protein structure is converted to a stick representation and then a fast filtering step is applied to find potential matches (using a bipartite matching algorithm), followed by a more exhaustive pairwise comparison between the filtered stick forms and the proteins (based on a double-dynamic programming algorithm and RMSD threshold for match set to 6 Å.)

By demonstrating the limitation on the number of realizable folding patterns, arising due to the restrictions imposed by the growth rules on feasible spatial assemblies of SSEs, the studies by Efimov (1997) and Taylor (2002) confirm the observations of Finkelstein and Ptitsyn (1987) and Chothia (1992). Moreover, these works inform new schemes to classify the observed protein folds (Gordeev et al., 2010).

Grishin and colleagues (Chitturi et al., 2016) recently proposed a method to enumerate constructively all idealized *parallel/antiparallel* arrangements of up to 5 SSEs. This work proposed a systematic enumeration of all possible parallel/antiparallel arrangements using a 3D lattice model. This allowed them to model theoretical arrangements of SSEs and use them to search for observed occurrences of each arrangement within the PDB. However, their idealized models are limited to parallel/antiparallel orientations, which poses a severe restriction in exploring the full set of SSE arrangements observed in the PDB.

Alva et al. (2015) sought regions of proteins that might comprise a set of ancestral fragments, conceivably vestiges of a pre-cellular “RNA-peptide world.” They identify 40 fragments, typically containing few secondary structure elements, that recur in many protein structures, including in sets of proteins not recognized as homologous. Some of these are similar to certain of our concepts; for instance, their set includes the standard supersecondary structures *α*-*α* hairpin, *β*-hairpin, and *β–α–β* unit. However, comprehensive coverage of observed protein folding patterns was not a goal of that work.

Other motif libraries have also been recently proposed: the Smotif library of Dybas and Fiser (2016) and the TERMs library of Mackenzie et al. (2016). An Smotif is designated by the arrangement of a *pair* of SSEs (of one of the following types: EE, EH, HE, and HH). A library of Smotifs is a collection of such SSE-pairs with different geometries. Their work utilizes an RMSD threshold of 2.5Å to cluster 11,068 observed pairs of SSEs from a collection of 1,200 protein structures (i.e., one randomly chosen protein domain per SCOP fold). These fragments serve in their work as the representatives of the protein structural space. Thus, any consecutive pair of secondary structures within a protein chain is assigned to the closest (based on RMSD) representative Smotif.

The tertiary motif (TERMs) library (Mackenzie et al., 2016) was able to find bigger assemblies of short oligopeptide fragments using the following approach. For each amino-acid residue *i* in the nonredundant collection of 29,000 residues, a candidate TERM is defined using one or more oligopeptide fragments formed by the union of the residues i−2,…,i+2 together with all penta-peptide regions around residues that form a “potential contact” with the residue *i*. For each candidate TERM, the method finds matching tertiary fragments using an RMSD-based search method. A subset of candidate TERMs is realized by posing it as the classical set cover problem and realizing the minimal cover using a greedy approximation method that iteratively identifies the TERMs (based on their coverage) that match proteins in the considered set. This iterative procedure yields about *half a million* (458,251) TERMs. The minimum TERM has 1 oligopeptide fragment containing 5 amino acids, whereas the maximum TERM has 10 fragments with 52 amino acids. Importantly, an average TERM in their library is composed of 3 oligopeptide fragments covering 19 amino acids (i.e., 6 amino acids per fragment). Furthermore, inspecting the TERMs that cover 50% of their proteins in their considered collection of 29,000 protein structures, we find that each TERM averages 2 fragments with 12 amino acids. Moreover, inspecting the top 24 TERMs [see Figure 2A of Mackenzie et al. (2016)], we find many repetitions of short helices and antiparallel strands.

Nepomnyachiy et al. (2017) recently proposed a pipeline to explore “reuse” of regions in proteins based on their amino acid sequence relationships. This work reported repeated occurrences of sequence segments between 35 and 200 amino acids in length. However, relying on amino-acid sequence relationships is rather limiting because sequences diverge more drastically than structures in evolution.

In comparison, our work results in only 1,493 architectural concepts (two orders of magnitude more concise than TERMs), where our smallest concepts contain 2 SSEs covering, on average, 19 amino acids—this is the median length of the regions where concepts with 2 SSEs are used, in the dissections of the structures from the PDB. The biggest concept is composed of 28 SSEs covering 171 amino acids. An average Proçodic concept in our dictionary is composed of 6 SSEs covering 75 amino acids. Considering the Proçodic concepts that cover 50% of the PDB, an average concept has 5 SSEs covering 66 amino acids. Thus, using this framework, our dictionary yields concepts that are a substantially larger than TERMs, and define a significantly more economical dictionary that explains the entire PDB. Moreover, the methodology we use defines a direct and efficient (dynamic-programming based) way to dissect any given protein structure using the inferred Proçodic dictionary.

These results are achieved due to the expressive power of tableaux to represent compactly the essence of protein folding patterns. This tableau representation, together with the minimum message length inference methodology, provides a reliable framework to compress without loss and identify relationships in the protein folding space.

## 3 Discussion

### 3.1 Many Concepts Are Linked to Ligand-Binding Sites

The molecular function of proteins is often mediated *via* interactions with chemical components such as metal ions, coenzymes, metabolic substrates, and nucleic acids, amongst others. Knowledge of such interactions is central to annotate protein function (Whisstock and Lesk, 2003; Goldstein, 2008), engineer new proteins (Gutteridge and Thornton, 2005), and design drugs (Rognan, 2007; Kinjo and Nakamura, 2009). These functionally critical interactions impose structural constraints on protein structures, as their domains evolve from a common ancestor. As noted by Lesk and Chothia (1980), in many cases active sites are the best-conserved regions within a family of protein structures (as seen in Figures 4, 5).

We have analyzed our dictionary and systematically identified concepts directly related to protein–ligand interactions. To achieve this, we mined and catalogued frequent ligand information (from “HETATM” records) derived from the source PDB entries of each concept usage (i.e., each instance in the PDB where the concept appears in the dissection of that protein’s tableau). Our definition of a *ligand* comes from the inventory of 23,258 chemical components specified by the LigandExpo (Feng et al., 2004) database. We note that this inventory does *not* exclude simple monovalent ions (such as $Na^{+},$ $K^{+}\text{, and }Cl^{-}$) or those that are often not biologically functional (such as sulfate $SO_{4}^{2-}$ ions). To complement this information, we also mined and cataloged keywords (from “KEYWDS” records) derived again from the source PDB entries of these concept usages. We used the observed frequencies of the bound ligands within the regions of concept usages, to narrow the initial set down to the 463 (31%) concepts that stand out in terms of recurrent patterns of interactions with the same set of ligand(s). These encompass interactions with monovalent ions, di-/tri-/tetra-valent ionic species, small molecules (including nucleotides), and macromolecular compounds, among others.

The fully annotated list of concepts with observed interactions with ligands/chemical components is available in the supporting data file: conceptsWithLigandInteractions.txt (click).

**FIGURE 6:**
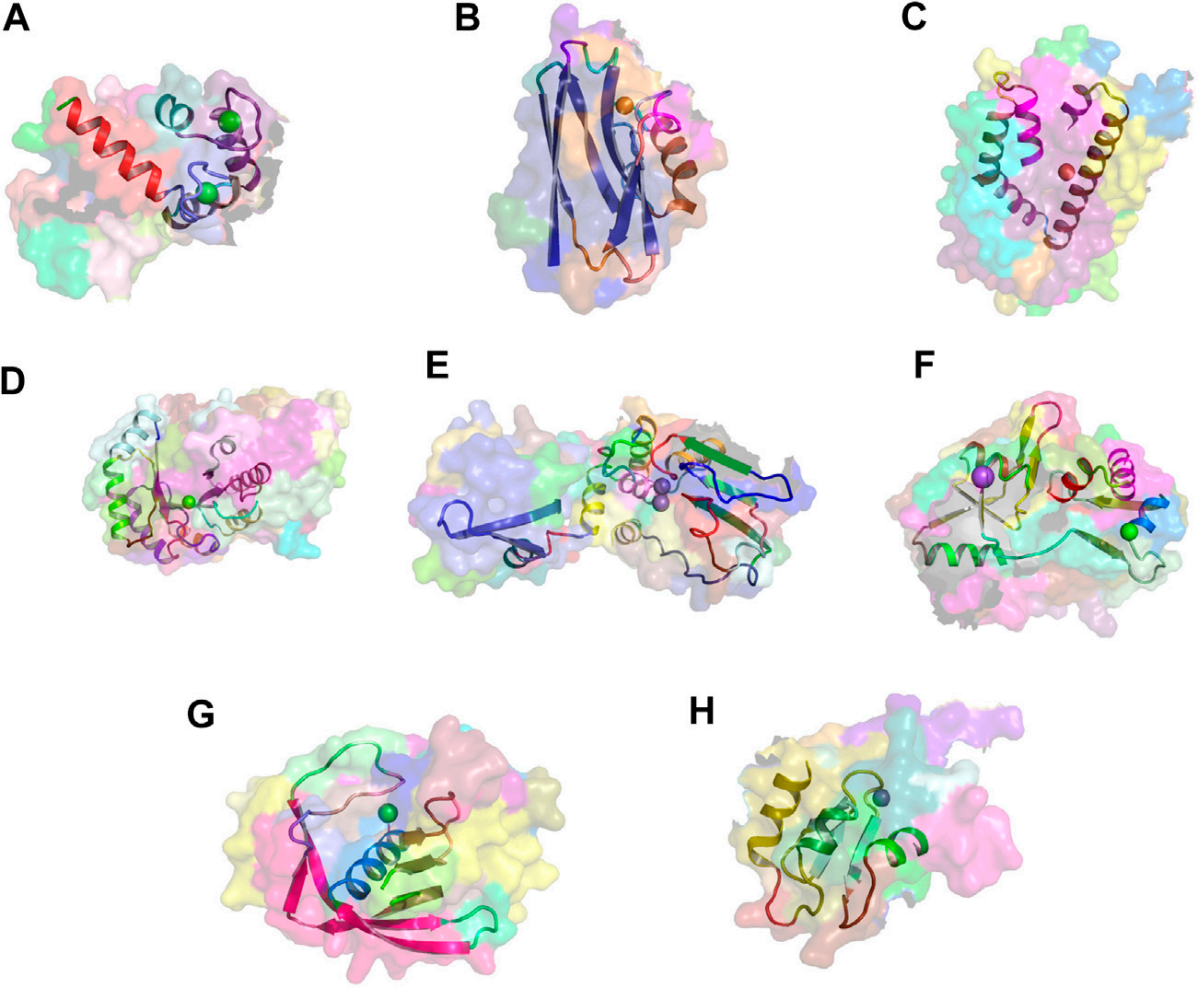
Metal-binding activity examples. Exemplars of usages of eight concepts linked to metal-binding activity. The region of concept usage is shown in cartoon in the context of the surface rendering of the source protein chain. **(A)** Usage of concept c_1099 within the calcium-bound calmodulin [1cdl (Meador et al., 1992)]. **(B)** Usage of concept c_432 within the copper-bound electron transfer protein [1a4b (Messerschmidt et al., 1998)]. **(C)** Usage of concept c_885 within the iron-bound oxidoreductase (2vux). **(D)** Usage of concept c_139 within the magnesium-bound lyase (3tte). **(E)** Usage of concept c_186 within the manganese-bound hydrolase [1k23 (Ahn et al., 2001)]. **(F)** Usage of concept c_133 within the sodium-bound Kainate and AMPA receptors [3g3g (Chaudhry et al., 2009)]. **(G)** Usage of concept c_280 within the nickel-bound peptide deformylase (2aia). **(H)** Usage of concept c_624 within the zinc-bound melanoma-inhibiting anti-apoptotic protein [1oy7 (Franklin et al., 2003)].

Figures 6A–G show examples of concept usages for a random selection of 8 concepts associated with metal-binding activity. Table 1 shows a partial list of concepts for which all (100%) of their usages show binding to the specified ligand/chemical components. Also shown are the extracted high-frequency keywords associated with usages of that concept, providing useful insights to impute functional roles. Among the shortlisted set of 463 concepts are those that demonstrably show binding specificity linked with target recognition, reception, and signaling (see Table 2).

**TABLE 1:** A partial list of concepts for which all (100% of) their usages show interactions with ligands or chemical components. This is derived by inspecting the ligand (“HETATM”) records within the source coordinate files of each concept usage. The bound ligands are shown (in the second column) using their standardized abbreviations, along with their observed frequency within the usages in parentheses. Also shown (in the third column) are the top keyword terms (from “KEYWDS” records specified by the structures’ authors) recurring within the usage coordinate files with their associated frequencies. (Note: CA = calcium ion).

Concept ID	Ligand/chemical component (freq)	Keyword (freq)
c_0011	PQQ (100%), CA (100%)	OXIDOREDUCTASE (90%), QUINOPROTEIN (27%)
c_0036	ZN (100%)	HYDROLASE (85%), EXOPEPTIDASE (46%), CARBOXYPEPTIDASE B (46%)
c_0065	FES (100%)	OXIDOREDUCTASE (96%), XANTHINE OXIDASE (32%), IRON SULFUR (30%)
c_0096	FMN (100%)	OXIDOREDUCTASE (100%), ROSSMANN FOLD (55%)
c_0108	HEM (100%), CA (100%)	OXIDOREDUCTASE (85%), PEROXIDASE (63%)
c_0110	HEM (100%)	OXIDOREDUCTASE (82%), MONOOXYGENASE (43%), CYTOCHROME P450 (34%)
c_0124	SF4 (100%), MG (100%)	OXIDOREDUCTASE (91%), [NIFE]HYDROGENASE (26%)
c_0144	CA (100%)	TRANSFERASE (81%), CGTASE (36%), ACARBOSE (33%)
c_0156	ZN (100%)	TRANSFERASE (90%), SET DOMAIN (39%), EPIGENETICS (28%)
c_0159	SF4 (100%)	OXIDOREDUCTASE (96%), NIFE HYDROGENASE (17%)
c_0208	CU (100%)	OXIDOREDUCTASE (97%), BETA BARREL (34%), LACCASE (32%)
c_0374	HEM (100%)	OXYGEN TRANSPORT (56%), HEMOGLOBIN (26%)
c_0397	ZN (100%)	OXIDOREDUCTASE (94%), SUPEROXIDE DISMUTASE (27%)
c_0424	PCA (100%)	HYDROLASE (95%), GLYCOSIDASE (35%), CELLULOSE DEGRADATION (32%)
c_0546	ZN (100%)	HYDROLASE (88%), PHOSPHODIESTERASE (32%), PDE (28%)
c_0568	FES (100%)	ELECTRON TRANSPORT (77%), FERREDOXIN (38%)
c_0604	HEM (100%)	ELECTRON TRANSPORT (100%), HEME (57%), CYTOCHROME B5 (40%)
c_0624	ZN (100%)	APOPTOSIS (47%), ZINC FINGER (44%), METAL BINDING (30%)
c_0714	NAG (100%)	VIRAL PROTEIN (84%), HEMAGGLUTININ (39%), GLYCOPROTEIN (22%)

**TABLE 2:** A partial list of concepts putatively linked to molecular reception, recognition, and signaling.

Concept ID	Ligand/chemical component (freq)	Frequent keywords (freq)
c_0062	NAG (96%), BMA (70%)	IMMUNE RECOGNITION (21%)
c_0133	ZN (35%)	AMPA RECEPTOR (26%), NEUROTRANSMITTER RECEPTOR (20%)
c_0205	GAL (36%)	CARBOHYDRATE RECOGNITION (11%)
c_0252	MYR (40%)	RHINOVIRUS COAT PROTEIN (20%), RECEPTOR (17%), ANTIVIRAL COMPOUND (10%)
c_0304	CA (60%)	ANTIBODY RECEPTOR (18%), CARBOHYDRATE RECOGNITION DOMAIN (15%)
c_0335	NAG (34%)	CELL ADHESION (29%), RECEPTOR (16%), GLYCOPROTEIN (11%)
c_0352	GOL (67%)	PEPTIDOGLYCAN RECOGNITION PROTEIN (10%)
c_0423	NAG (63%)	IMMUNE SYSTEM (87%), ANTIGEN PRESENTATION (26%), T CELL RECEPTOR (12%)
c_0572	FMN (67%)	PHOTORECEPTOR (36%), LIGHT-INDUCED SIGNAL TRANSDUCTION (13%)
c_0819	ZN (58%)	SIGNALING PROTEIN (19%), PHOTORECEPTOR (13%)

The full list of inferred concepts putatively linked to molecular reception, recognition, and signaling is available in the supporting data file: receptorConcepts.pdf (click).

### 3.2 Inferring Biological Function From Concept Usage Information

Many proteins are deposited into the PDB with unspecified functional annotation, especially those coming from structural genomic initiatives. Functional characterization of such proteins is of crucial importance to the structural biology community. Its importance can be evidenced by the community-wide Critical Assessment of protein Function Annotation program (CAFA, biofunctionprediction.org/cafa/), which assesses methods dedicated to predicting protein function from an amino-acid sequence.

**FIGURE 7:**
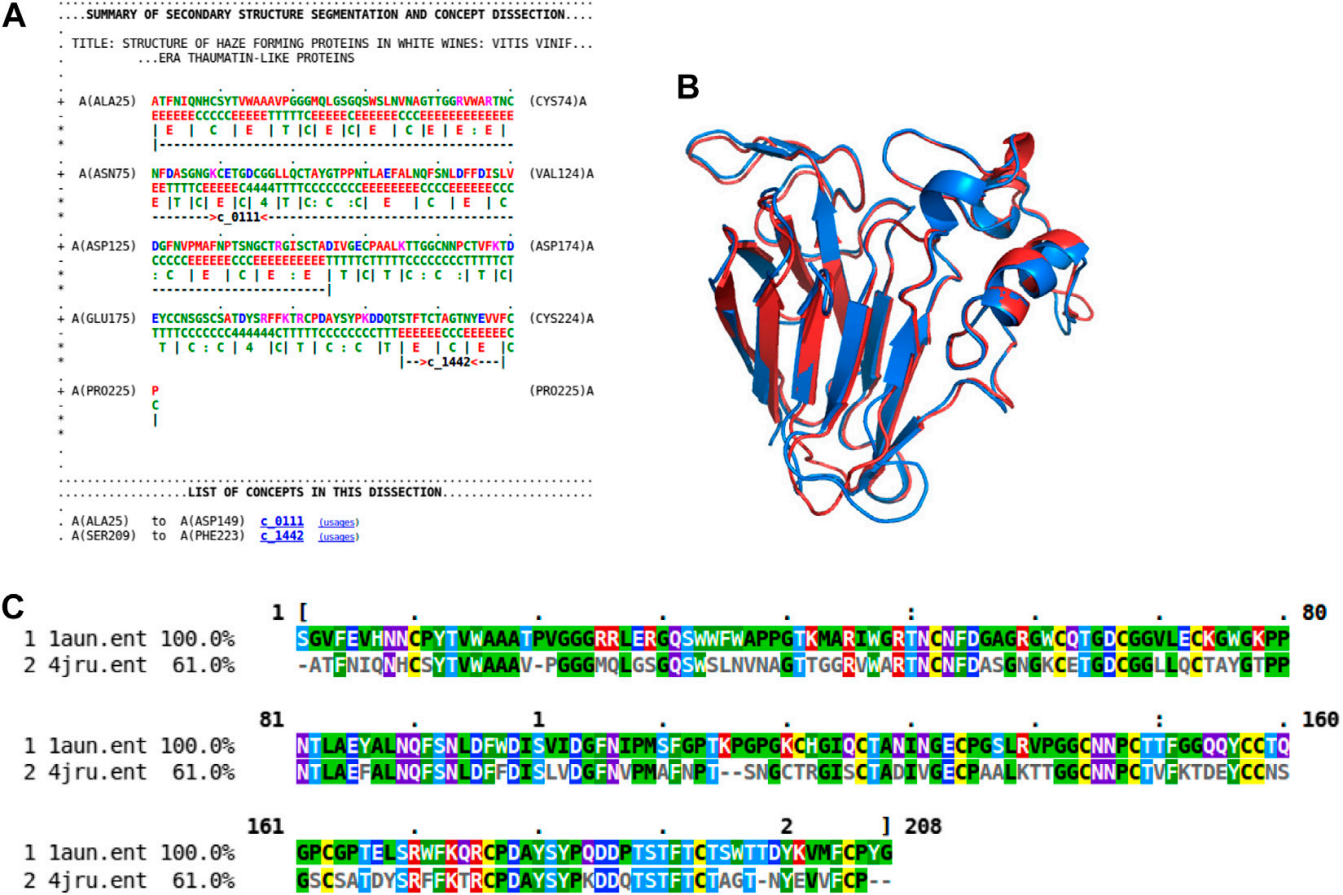
Dissection case-study on the haze-forming thaumatin-like protein, 4jru. **(A)** Dissection output from Proçodic of the haze-forming thaumatin-like protein in white wines from *Vitis vinifera* (4jru). **(B)** Superposition of this haze-forming protein and the pathogenesis-related PR-5d protein of tobacco (*Nicotiana tabacum*; 1aun). 4jru is shown in blue; 1aun is shown in red. This superposition was based on the structural alignment produced by MMLigner (Collier et al., 2017), which is shown in **(C)**.

As previously shown (Figure 4A), the rich source of information within this concept dictionary is useful to investigate and impute biological function. More evidence of this is shown by another case study involving the haze-forming thaumatin-like protein in white wines made from *Vitis vinifera* (4jru containing 201 residues). Figure 7 gives the dissection of 4jru composed of two concepts c_0111 and c_1442.

Concept c_1442 is of less functional interest as it defines a common *β*-hairpin unit consisting of two antiparallel *β*-strands. On the other hand, c_0111 contains 12 strands that assemble to form mainly two face-to-face packed antiparallel *β*-sheets with an extended *β*-ribbon connected by an Ω-loop (Leszczynski and Rose, 1986). This multistranded motif is characteristic of thaumatin-like proteins (Ogata et al., 1992). Examining the usages of this concept within the PDB *via* our Proçodic web site, we find it is used at 15 other loci, most of them thaumatin/osmatin-like proteins, with their top two keywords displaying “antifungal protein (53.3%)” and “plant protein (46.7%),” respectively. Figures 7B,C show the structural alignment of 4jru with the usage in the pathogenesis-related PR-5D protein of tobacco (*Nicotiana tabacum*; 1aun with 208 residues) that results in a superposition with 1.47 Å root-mean-square deviation over 201 amino-acid residues between the C_*α*_ coordinates of the two structures. This specific PR-5D protein is classified functionally as an antifungal protein, and, in general, proteins of this class have known pathogenesis-related antifungal activity. This suggests that the haze-forming protein might exhibit the same biological function.

In some cases, the information provided by this dictionary can lead to a reliable but less-specific functional classification prediction, for example putatively identifying a general type of function such as “oxidoreductase” or “lyase.” Such generic functional classification can be useful, as it may provide guidance for laboratory experiments aimed at defining the function more precisely, especially if clues about a ligand-binding site are available. For example, consider the crystal structure of dihydrodipicolinate synthase (DapA) from *Agrobacterium tumefaciens* (2hmc). The dissection of this DapA structure shows the usage of concept c_0008 covering its entire chain A. About 90% of c_0008’s 118 usages show the functional classification as “lyase.” DapA belong to the family of amine-lyases that catalyze the cleaving of carbon–nitrogen bonds, playing an important role in lysine biosynthesis in prokaryotes, phycomycetes, and plants (Mirwaldt et al., 1995).

### 3.3 Local Sequence–Structure Correlation Within Concept Usages

The identification of structural features that have strong amino-acid sequence preferences is central to structure prediction (Bystroff and Baker, 1998). Therefore, we studied the concept usages within the PDB to explore the conformational preferences of local sequences. To achieve this, for each concept, the amino-acid sequences in the regions of concept usages within the PDB were extracted, and the sequences in each set were aligned and clustered (Sievers and Higgins, 2014).

Almost 20% of the concepts in our dictionary (288 out of 1,493) have associated amino-acid sequence patterns that cluster into a single group (Supplementary Figure S4A). When considering the (normalized) ratio of clusters over the number of *nonidentical* amino-acid sequences of concept usages (Supplementary Figure S4B), almost 30% of the concepts (441) have a ratio smaller than 0.05, whereas almost 50% (738) have a ratio between 0.05 and 0.1. Together, this indicates that for almost 80% of the concepts in our dictionary, their usages of amino-acid sequences cluster into a small number of groups (<10% of their total unique amino-acid sequences).

**FIGURE 8:**
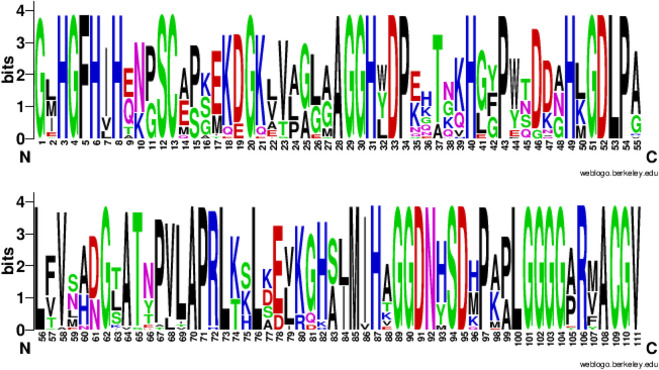
Sequence consensus across the usages of concept c_0397. amino-acid sequence logo (in two parts: columns 1-55 and 56-111) showing the sequence consensus across the usages of a randomly chosen concept c_0397 directly related to the Cu-Zn binding superoxide dismutase. Of the 111 columns in the multiple sequence alignment (of the c_0397’s 33 usage sequences) corresponding to this logo, 46 aligned columns show a consensus of 100%.

This strong sequence dependence is expected, particularly for concepts linked to ligand binding or other functional units. For example, Figure 8 shows the sequence logo obtained from the multiple sequence alignment of the usage sequences of the concept c_0397. This concept is related to the Cu-Zn type I (SODI) superoxide dismutase, which has a β−barrel-like subunit with copper and zinc ions bound at the active site. This is common in many Gram-negative bacterial pathogens (amongst others) to counteract a burst of toxic superoxide radicals under oxidative stress (Forest et al., 2000).

There is a potential application of the observed sequence–structure correlations to structure prediction. We downloaded the coordinate files of 33 PDB structures specified in the description field of the CASP12 target list available at http://predictioncenter.org/casp12/targetlist.cgi. Each chain from these 33 structures was independently dissected using the Proçodic dictionary of concepts. The dissection of protein chains defines nonoverlapping regions assigned either to one of the dictionary concepts (c_0001 – c_1493), or a *null* concept (c_0000). For each region assigned to a dictionary concept, we extracted the associated target amino-acid sequence and performed a pairwise *sequence* alignment with each of the local amino-acid sequences defined by the concept usages. This exercise identified a subset of concept usages in the PDB whose local amino-acid sequences have a detectable similarity with the target. Table 3 quantifies the extent of coverage of these regions for each of the 33 CASP12 targets. This table shows that in 26 of 33 cases, more than 50% of the target amino-acid chain has detectable sequence similarity that can be derived from the usage information.

**TABLE 3:** Statistics showing the extent of detectable sequence similarity on each of the 33 CASP12 targets with their PDBIDs specified at http://predictioncenter.org/casp12/targetlist.cgi. First column: PDBID of the 3D experimental structure of each CASP12 target. Second column: The coverage statistics in terms of the total number of amino acids (#a.a.) within the amino acid (sub-)sequences defined by the dissected regions of the target protein with detectable sequence similarity with amino acid (sub-)sequences of their corresponding concept usage instances (see the main text). Third column: The total number of amino acids in the target protein, cumulative over all chains. Fourth column: Percentage coverage = Second column*100/Third column.

Target’s	#a.a.’s in regions	Total #a.a.’s	
PDBID	with usage seq hits	in all chains	%Covered
3JB5	1046	2076	50.4
4YMP	202	215	94.0
5A7D	4468	5065	88.2
5AOT	91	102	89.2
5AOZ	125	141	88.7
5D9G	154	502	30.7
5ERE	417	540	77.2
5FHY	155	458	33.8
5FJL	88	136	64.7
5G3Q	100	168	59.5
5G5N	580	1022	56.8
5HKQ	160	263	60.8
5IDJ	63	242	26.0
5J4A	339	440	77.0
5J5V	815	1065	76.5
5JMB	103	182	56.6
5JMU	172	219	78.5
5JO9	215	239	90.0
5JZR	203	262	77.5
5KKP	166	509	32.6
5KO9	73	253	28.9
5LEV	323	375	86.1
5M2O	171	211	81.0
5MQP	2674	4801	55.7
5NSJ	150	284	52.8
5NV4	713	1377	51.8
5SY1	786	1458	53.9
5T87	444	745	59.6
5TF2	331	338	97.9
5TJ4	2640	5462	48.3
5UNB	378	681	55.5
5UVN	954	2496	38.2
5UW2	211	332	63.6

It should be noted that we used the structural information of CASP12 targets to dissect the protein chains, before identifying the sequence relationships of the target sequence and those within the concept usages. However, for the proper application to structure prediction, the identification of sequence hits with concept usages should be carried out using only the target sequence. In principle, this can be done by sliding along the target sequence with varying window sizes, and exploring the sequence similarity with the sequences across all usages of every concept in the dictionary. Nevertheless, this preliminary analysis can be used to hypothesize reasonably that these local sequence–structure relationships provide a strong potential to support structure prediction efforts, especially since an average concept usage spans significantly longer stretches along the protein chain than the currently considered oligopeptide-fragment libraries used by fragment-based *ab initio* protein modeling approaches. Thus, this information can be potentially utilized to model several nonoverlapping regions in the target protein chains by the structure-prediction servers (Kim et al., 2004; Källberg et al., 2012; Waterhouse et al., 2018; Zheng et al., 2019; Senior et al., 2020).

As a note on the latest breakthrough in the field of structure prediction, convolutional neural network-based prediction architectures [especially AlphaFold (Senior et al., 2020)] have seen groundbreaking success in the CASP13 and CASP14 rounds. These neural network methods train on multiple sequence alignments as inputs, involving either whole or part of the target sequence whose structure is being predicted. At the time of writing this article, the technical details of the AlphaFold system used in CASP14 remain unpublished. When these details become open, it would be useful to explore whether sequence–structure correlations at the level of concepts can be incorporated into training the neural networks more efficiently—as per the information disclosed by Google Deepmind the current architecture requires in the order of 128 Tensor Processing Units and over a few weeks to predict structure from sequence, but with groundbreaking accuracy.

The amino-acid subsequences of nonoverlapping regions dissected using the Proçodic dictionary of concepts are available at: casp12_prosodic_dissections.tgz (click). The information of dissected target region followed by other subsequences in the usages of the corresponding (assigned) concept with demonstrable sequence similarity (under pairwise sequence alignment with the target subsequence) is available at: casp12_concept_usage_hits.tgz (click). The multiple sequence alignments [using MUSCLE (Edgar, 2004) with default options] of the identified sequence hits are available at: casp12_concept_usage_hits_msa.tgz (click).

### 3.4 Exploration of Substructures and Structural Relationships

In addition to the applications explored above, the dictionary can be used to complement standard protein structural studies. Researchers can approach the dictionary with a particular structure or family of structures in mind. For example, dissecting the human hemoglobin (1hho, chain A) at the Proçodic website identifies the concepts c_0375, c_0894, and c_1410. Choosing one of the concepts, for example, c_0894, its archetype is found in d1x9fd, a globin from the annelid *Lumbricus terrestris*. Note that related proteins can present dissections into different concepts. However, these concepts may still be related (see Section 3 on hierarchical clustering of concepts). Our dictionary subsumes known supersecondary structural motifs. For example, c_0375 and c_0894 are related concepts linked to globins, with the former being more elaborate (with three extra helices) than the latter. Examining the corresponding concept “usages” link on the Proçodic website reveals that many usages of these related concepts appear in other globins. Supplementary Material S1 contains several examples of use of Proçodic to explore protein substructural similarities.

## 4 Materials and Methods

### 4.1 Tableau Representation

Tableaux are concise two-dimensional representations of protein folding patterns (Lesk, 1995). A tableau represents a protein folding pattern in terms of a) the order of secondary structural elements that appear along the polypeptide chain, and b) the geometry of interactions of pairs of SSEs in contact. This provides a computable definition for protein folding patterns, useful to study many aspects of protein architecture (Kamat and Lesk, 2007; Konagurthu et al., 2008; Konagurthu and Lesk, 2010).

For a protein of known 3D structure, the construction of a tableau involves first assigning the SSEs from the set of coordinates. In this work, we assign the secondary structure using the program SST (Konagurthu et al., 2012). This identifies the order in which helices (H) and strands of sheet (E) appear along the polypeptide chain. The succession of SSEs in any protein therefore appears as a string of characters H or E. The relative orientation of each pair of SSEs is computed as a dihedral angle between two planes formed by the least-squares vectors fitting the C*α* coordinates of each SSE (directed from N- to C-terminus) and their mutual perpendicular.

The geometry of pairs of SSEs is represented as a square-symmetric matrix of orientation angles, with rows and columns indexed by successive SSEs. A corresponding contact matrix stores the contact patterns between pairs of SSEs. Two SSEs are said to be in contact if there exists at least a pair of residues (one from each SSE) that are in contact. Two residues are in contact if there is at least one pair of atoms (one from each residue) the distance between which is less than the sum of their Van der Waals radii plus a small constant (1 Å).

The idea is that the essence of a protein folding pattern is contained in the SSEs, their contact patterns, and the relative orientations of pairs of SSEs in contact.

### 4.2 Source Collection Used for the Inference of Proçodic Dictionary of Concepts

A *source collection* is a collection of (source) tableaux T. Since the full PDB has redundancy and bias in terms of entries with similar structures, to infer the dictionary of concepts we use the ASTRAL SCOP-95 (Murzin et al., 1995; Andreeva et al., 2013; Chandonia et al., 2017) (v2.05) dataset that has been produced to remove bias due to over-represented structures, while explicitly incorporating structure quality at each step of the domain selection (Brenner et al., 2000). This data set is composed of 26,949 domains, representing only 12% of the full SCOPe (v2.05) domain dataset. Of these, 13,365 domains have < 40% sequence similarity to its closest neighbor. Although the maximum sequence similarity two proteins can share is 95%, the average sequence similarity is significantly lower (< 53%). The full list of ASTRAL SCOP-95 domains used to infer the reported dictionary is available in the supporting data file: prosodicInferenceList.txt (click). Further, the inferred Proçodic dictionary was used to dissect the PDB (Berman et al., 2003). Analysis presented here includes the dissections of 113,724 protein coordinates files: prosodicDissectedWWPDBList.txt (click). In addition to these dissections, the Proçodic website allows users interactively to dissect any protein structure on demand.

### 4.3 Definitions of a Concept and a Dictionary of Concepts

Any subtableau comprising ≥ 2 consecutive rows and columns is potentially a concept, provided that the graph defined by the corresponding contact matrix is connected. (An undirected graph is said to be connected if there exists a path between any pair of vertices.) The rationale for this definition is supported by the analysis by Kamat and Lesk (2007) who demonstrated that almost all the information required to identify a folding pattern is inherent in the local structure, which can be captured using successive diagonals of a tableau. We also note that relaxing the concept definition to general subtableaux with nonconsecutive SSEs would render the problem of finding an optimal set of concepts computationally intractable.

A candidate *dictionary* C is a set of concepts. Any possible dictionary is a set of substructures, each satisfying the definition of a concept, that appear in the source collection. Our goal is to determine the optimal concept dictionary to *explain* the entire source collection as efficiently as possible. Technically, this is the dictionary that gives the most (lossless) compression of the source collection.

Associated with each concept c∈C is a concentration parameter, *κ*, corresponding to a von Mises circular (angular) probability distribution (Mardia and Jupp, 2009). This parameter controls the assignment of probabilities used to estimate the encoding length of entries in Ω when compressing regions of the source tableaux. That is, *κ* controls the *flexibility* of an inferred concept. A smaller/larger $\kappa_{y}$ yields greater/lesser flexibility of the concept’s usages for compressing source tableaux regions. These values are inferred as a part of the dictionary search (see Algorithm 1).

### 4.4 Inference of Dictionary of Concepts

We recently described a lossless compression-based methodology to infer recurrent subtableaux on any source collection of tableaux using the Minimum Message Length (MML) criterion (Subramanian et al., 2017). The dictionary we report here has been subsequently inferred using the methodology described in that work. For convenience of the reader, the overview of our methodology is summarized later, and we refer the reader to our published methodology (Subramanian et al., 2017) for formal details.

The main goal here is to learn a flat (nonhierarchical) dictionary of concepts C that yields the best lossless compression of the source collection of tableaux T. The inference of C was undertaken using the Bayesian criterion of minimum message length (MML) (Wallace, 2005). MML provides a statistical inference framework to learn propositions from any observed data set. A proposition can be made as a hypothesis, model, explanation, or theory (Allison, 2018). The MML framework combines ideas from the field of information theory developed by Shannon (Shannon, 1948) and Bayesian inference. Using MML, the descriptive complexity of any stated hypothesis (model, theory, etc.) and its fidelity to explain the observed data can be accurately quantified in terms of Shannon information content (measured in *bits*). This allows the MML framework to provide a reliable complexity-versus-fidelity trade-off, and overcome the well-known problem of over-fitting that is observed in many statistical inference problems. Thus, the best hypothesis is chosen to be the one that yields the most succinct two-part encoding, where the first part encodes the hypothesis, whereas the second encodes the observed data *given* the stated hypothesis. From the Bayesian standpoint (Bayes and Price, 1763), this translates to finding the hypothesis on the data that maximizes their joint probability. Applying MML to this work, the best concept-dictionary C that explains a source collection of tableaux T is the one that minimizes the length of the two-part encoding of the form: ℐ(C&T)=ℐ(C)+ℐ(T|C), where $ℐ\left(\cdot \right)=-{\text{log}}_{2}\left(\text{Pr}\left(\cdot \right)\right)$ measures the Shannon information content (Shannon, 1948) of each of the two parts.

The MML framework provides a natural null-hypothesis test. A dictionary C explaining T is accepted if and only if its two-part lossless encoding length is *shorter* than the encoding length of the observed data communicated independently (without the support of any dictionary). The latter is termed the null model message length and denoted as ${ℐ}_{\text{null}}\left(\cdot \right)$. Further, the quality of an inferred dictionary can be measured by the amount of lossless compression gained with respect to the null model: ${ℐ}_{\text{null}}\left(T\right)-ℐ\left(C\&T\right)$. Thus, using MML, the best dictionary can be equivalently chosen using the following objective: $\underset{C}{\arg\;\max}\;{ℐ}_{null}\left(T\right)-ℐ\left(C\&T\right)$. Formal details of the search methodology for the best dictionary and MML methods to estimate the lossless encoding lengths given by the terms ${ℐ}_{\text{null}}\left(T\right)$ and ℐ(C&T) appear in (Subramanian et al., 2017).

Broadly speaking, central to the inference of the dictionary is the procedure to generate the optimal encoding of any single tableau using a given concept-dictionary C. This involves: 1) the optimal partitioning of the tableau into subtableaux, 2) the optimal assignment of those regions to concepts in the dictionary (or to a null concept), and 3) the encoding of information within the whole tableau using the assignment of regions to their respective concepts. This optimal partitioning and encoding is chosen as the one that yields the minimum encoding length, and can be derived using an efficient dynamic programming algorithm. Therefore, using MML, the best dictionary for any source collection of tableaux is defined as the one that yields the shortest overall encoding of stating the dictionary, plus the optimal encodings for each tableau in the collection given that dictionary.

**Algorithm 1:** Simulated annealing algorithm to find the optimal dictionary.

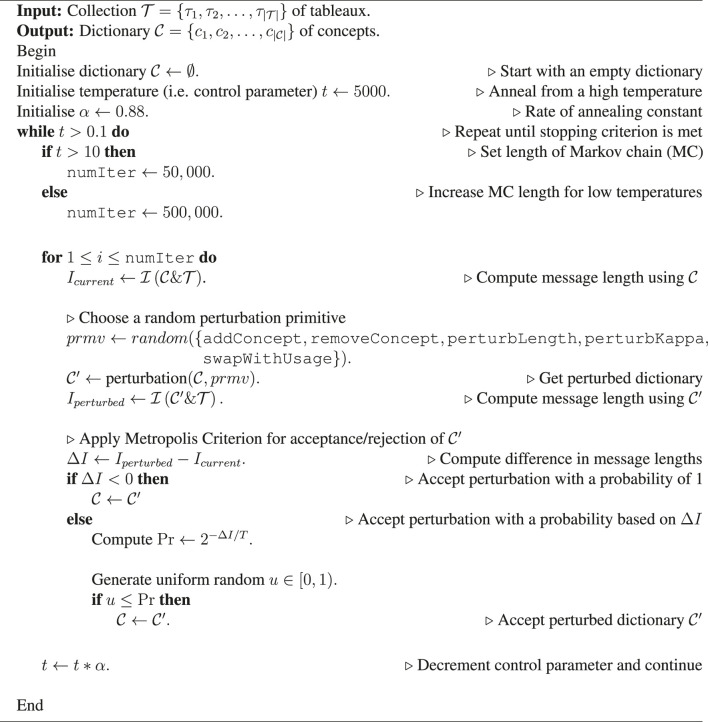

Finally, the search for the best dictionary is carried out using simulated annealing (see Algorithm 1). Starting from the initial state of an *empty* dictionary, the search involves iterative and stochastic explorations of local neighborhood of the solution-space using the following perturbation primitives: 1) Add concept: randomly choose a subtableau (candidate concept) from the source collection and add it to the current dictionary. 2) Remove concept: randomly choose and delete an existing concept from the current dictionary. 3) Perturb concept length: randomly choose an existing concept from the dictionary and extend/shorten it by one SSE, with reference to the concept’s original source (in the source collection). 4) Perturb kappa: randomly choose a concept and perturb its statistical parameter (*κ*) that controls its flexibility. 5) Swap concept with usage: randomly choose a concept from the current dictionary, and swap it with a region in the collection that is currently encoded by it.

## Data Availability

The original contributions presented in the study are included in the article/Supplementary Material. Further inquiries can be directed to the corresponding author.
