# Supplementary material for: Universal Architectural Concepts Underlying Protein Folding Patterns
Source: Front Mol Biosci. 2021 Apr 30;7:612920. doi: 10.3389/fmolb.2020.612920 (PMC8120156; doi:10.3389/fmolb.2020.612920)
Supplement: Supplementary file 1 [file DataSheet1.PDF]

Supporting material for ‘*Universal architectural concepts underlying protein folding patterns*’

Arun S. Konagurthu[a,*], Ramanan Subramanian[a], Lloyd Allison[a], David Abramson[b], Peter J. Stuckey[a,c], Maria Garcia de la Banda[a], and Arthur M. Lesk[d,e,*]

[a]Department of Data Science and Artificial Intelligence, Faculty of Information Technology, Monash University, Clayton, VIC 3800, Australia; [b]Research Computing Center, University of Queensland, Brisbane, QLD 4072, Australia; [c]School of Computing and Information Systems, University of Melbourne, VIC 3010, Australia; [d]Department of Biochemistry and Molecular Biology, Pennsylvania State University, University Park, PA 16802, U.S.A.; [e]MRC Laboratory of Molecular Biology, Francis Crick Avenue, Cambridge CB2 0QH, U.K.

PROÇODIC website: An interactive website describing the inferred concepts and associated information is available at: http://lcb.infotech.monash.edu.au/prosodic/

S1. Supplementary Notes for ‘Exploration of substructures and structural relationships’

Below we demonstrate with examples the use of PROÇODIC to explore relationships between proteins at the level of substructures.

Globins. As seen in the main text, two related related proteins can present dissections into different related concepts; this is the result of the calculation to optimize the representation of the whole set of proteins. For closely-related proteins, the three-dimensional structures of the usage instances of a given concept are superposable. Fig. SF1(a) shows the superposition of the instances of c_0894 from the α subunits of human deoxyhaemoglobin (2DN2), and oxyhaemoglobin from the common pigeon *(Columba livia)* (2R80). In the structural superposition, 78 Cα atoms from these regions fit to an r.m.s.d. of 0.83 Å.

More distantly-related globins may also share the concept but the regions are not so precisely superposable. For example, a structurally-diverged class of globins are the truncated globins, substantially shorter than sperm-whale myoglobin and showing substantial structural changes. Fig. SF1(b) shows the superposition of the instances of c_0894 from human oxyhaemoglobin (1HHO) and the truncated globin from the ciliate *Tetrahymena pyriformis* (3AQ5). Note that the helix lengths are much more variable than in the superposition shown in Fig. SF1(a). The loop region at the top of this figure does not superpose well between the two structures, and indeed does not even have the same length. This emphasises that our representation of folding patterns captured via the subtableaux of concepts is at the level of geometry of secondary structure elements.

The list of wwPDB entries reported from c_0894’s ‘usage’ link contains proteins identified as non-globins, for instance complex II (succinate dehydrogenase) from *Escherichia coli,* a membrane protein, (1NEN chain B residues ASP144–LEU197). Superposing the residues in this region with the instance in 1HHO gives the result shown in Fig. SF1(c). The helices from the N and C termini of these regions fit well. The intervening region shares the secondary structure with some conformational differences.

Could it be that *Escherichia coli* complex II (succinate dehydrogenase) is really homologous to the globins? Examination of 1NEN shows that this protein includes strands of β-sheet, ruling out the possibility of similar topologies of their overall folding patterns.

* Correspondence: aml25@psu.edu or arun.konagurthu@monash.edu

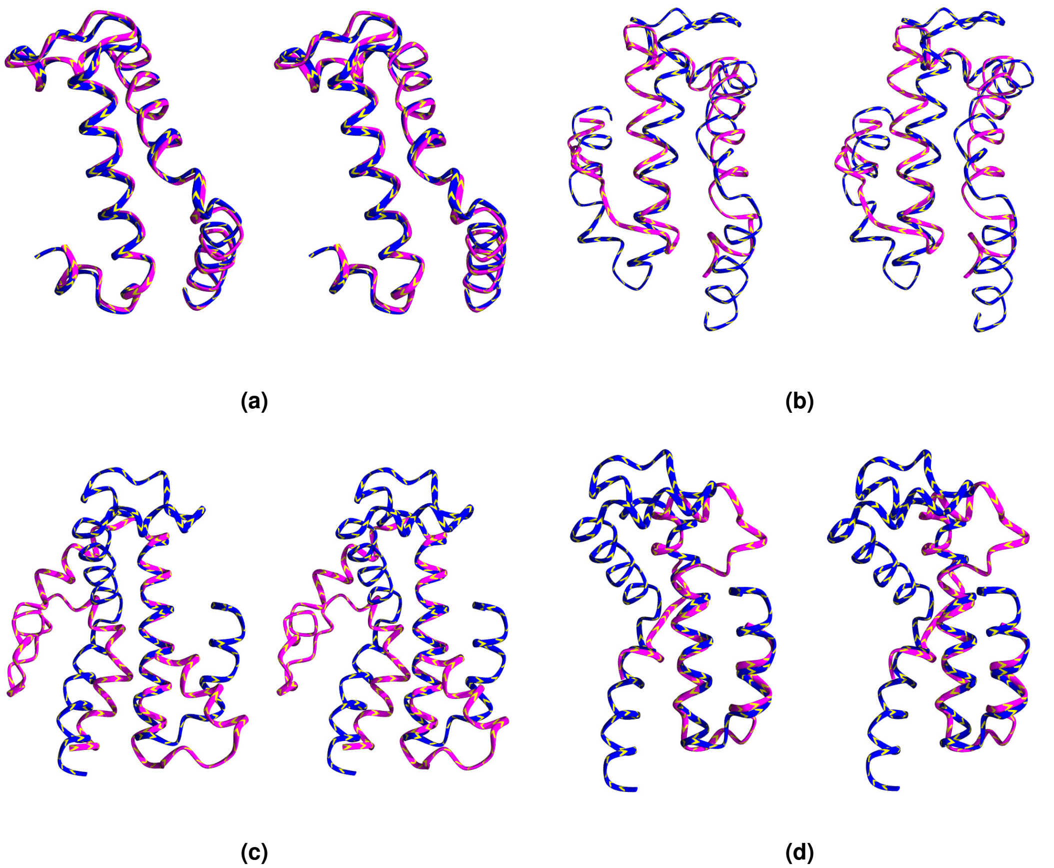

Fig. SF1. Exploring relationships among Globins.
Superposition of the usage instances of concept `c_0894` (shown in stereo). (a) Superposition of instances from the α chains of human deoxyhaemoglobin (blue) and oxyhaemoglobin from common pigeon *(Columba livia)* 2R80 (pink). (b) Superposition of instances from human oxyhaemoglobin (1hho) and the truncated globin of *Tetrahymena pyriformis* 3AQ5. (c) Superposition of instances from human haemoglobin (1HHO chain A) with the corresponding usages in an *unrelated* protein, complex II (succinate dehydrogenase) from *E. coli* (1NEN chain B). (d) Superposition of 1HHO with unrelated human squalene synthase (3VJ8).

Another example that shares a concept with human haemoglobin, is the human squalene synthase (3VJ8). (Fig. SF1(c)) Comparing the superpositions within the globin family with the superpositions involving globins and non-globins, closely-related globins show a well-fitting superposition of all secondary structures using the same overall rotation and translation, but globin–non-globin superpositions do not. This is because preserving the angles between successive secondary structures does not fix the global structure, although it does constrain it.

TIM-barrels. When probing the web site for a standard type of structure, the TIM barrel, we enter into the keyword window the string `EHEHEHEHEHEHEHEH` – or its regular expression: `(EH){8}` – signifying an eight-fold repeat of a β-α unit.

The web site returned two concepts, `c_0008` and `c_0032` containing the pattern `EHEHEHEHEHEHEHEH`. The first, `c_0008`, contains TIM barrels. The structure of the concept comprises the canonical 8-fold β-α barrel plus four additional C-terminal helices. As the reader is encouraged to try, clicking on the image produces a large 'still' high-quality graphic display; clicking on 'view interactively' produces an image rotatable under mouse control. From the main listing of all dictionary concepts on the website, clicking 'full details' gives the full secondary-structure assignment for the 'fold archetype' of `c_0008`, SCOP domain `d3flua_`, and the tableau computed for this domain. (Our methodology enables each concept in the dictionary to converge to an archetype that can viewed as the topological *median* over all usages of that concepts in the source collection the dictionary compresses.) Varying ideas defining such topological medians representing supersecondary structural motifs were previously explored, especially as 'attractors in fold space' to enable protein structural classification efforts (1, 2).

Clicking on 'usages' reports other instances of this concept in the `wwPDB`. There are 118 other usages, all TIM barrels. However, they are not the only TIM barrels in the `wwPDB`. Many structures that do contain the 8-fold barrel but lack the four C-terminal helices are dissected into smaller units, some containing β-α subsets of the barrel. Others proteins have helices inserted at different points, deviating from the specified secondary structural pattern. Therefore, searching using patterns alone does not return all the TIM barrels in the `wwPDB`.

It is possible to type 'TIM barrel' into the keyword field. The web site will then return many concepts, some of which do correspond to TIM barrels, but others of which do not. For instance, in response to the keyword query 'TIM barrel' PROÇODIC returns `c_0004` which contains 24 consecutive β-strands but no helices. The reason is that one of the `wwPDB` entries in which `c_0004` appears is the human PRMT5:MEP50 complex (4QGB): this entry protein does contain a TIM-barrel domain (in which `c_0004` does not appear), and the entry file contains TIM BARREL in its `wwPDB` KEYWDS record line, which triggers a hit on this concept. In summary, to find TIM barrels in the `wwPDB`, a PROÇODIC search for (EH){8} returns too little, a search for 'TIM BARREL' returns too much, and there is no Goldilocks compromise. In any event, this is a solved problem. For this particular question, other tools such as SCOP are more convenient and appropriate.

The other concept returned for the query `EHEHEHEHEHEHEHEHEH`, contains two domains, each with four β-α units, but not closed into a barrel. This appears because a sequence of secondary structure elements does not uniquely define three-dimensional structure.

Uncompressed regions in dissections and unusual structural components. Suppose a region in a protein contains an unusual conformation. It may require a shorter message to send the subtableau information of

this region raw (without compressing that region) than to include a representative within the dictionary and compress it. This is because, given its rarity, the overhead of adding it in the dictionary does not justify its inclusion as a separate concept. Overall, in the dissections of ∼114 000 wwPDB entries, ∼66% of the residues are covered by dictionary concepts.

An example of an uncompressed region appears in the dissection of *Chironomus* erythrocruorin, a globin from an insect. Although the overall structure of this molecule is similar to that of globins, there are deviations from the usual structure in the region corresponding to the D helix of sperm whale myoglobin. This results in different dissections of sperm whale myoglobin (1MBD) and *Chironomus* erythrocruorin (1ECD):

Sperm whale myoglobin (1MBD):		*Chironomus* erythrocruorin (1ECD):	
SER3 – PRO37	c_1483	ALA3 – ASP31	c_1368
PRO37 – LYS79	c_1141	ALA53 – MET136	c_1140
HIS82 – LEU149	c_1433		

Observe that the region in *Chironomus* erythrocruorin from residues 31–53 is not part of the dissection. This region is explained directly without compression, using what we call the *null* concept (3).

Erythrocruorins. Typing 'erythrocruorin' in the keyword field returns 26 concepts. The top one, concept c_0547, is an assembly of seven helices, corresponding to helices A-B-C-E-F-G-H in the canonical globin fold. There are 268 usage instances of this concept, which include erythrocuorins and globins. (The seven helix pattern fits the $\alpha-$chain of mammalian haemoglobins, which lack a D helix, but not the β-chain which contains a D helix.) The distinction between similar structures that differ in some detail which breaks a pattern, can be seen as both a strength and as a weakness. We saw a similar phenomenon with the TIM barrels and with globins.

Substructures of the 'globin fold' containing 6 helices, include c_0640. This corresponds to globin helices A-B-E-F-G-H. Examples include phycocyanins and phycoerythrins, colicin, and certain globins (4). To be a proper instance of c_0640, a globin must *lack* both C and D helices. In these cases the region corresponding to the D helix is not helical, but the region corresponding to the C-helix is nevertheless quite close to the expected 3_{10} helix. This is because the region is distorted enough to drag at least one hydrogen bond outside the thresholds of acceptance in distance and/or angle. As a result, the region is assigned as a coil, and only six helices are attributed to the chain. In other cases, the compression criterion encodes a regular globin structure as more than a single concept. Thus, human oxyhaemoglobin (1HHO), chain A, is decomposed into:

SER3 – ARG92	c_0894
VAL96 – ARG141	c_1410

Note that this chain does have a 3_{10} C helix, but *not* a D helix.

In addition to helical concepts that are substructures of the canonical globin fold, querying for erythrocruorins (as a keyword on the web site) returns concepts containing purely β-sheet concepts. These are known to appear in large, multimeric, extracellular invertebrate erythrocruorins, as linker regions between globin-like all-helical domains (5). Our dictionary has therefore called for attention to these additional substructures, not customary structure components from the familiar globin family.

As a β-sheet structure is rare in the globins and erythrocruorins, it is of interest to explore the relationships of these substructures to other families. Are there homologues, of which the erythrocruorin linker domain might be part of a chain of evolutionary relationships?

Consider the concept c_0559, comprising six β-strands. Its *usages* provides a list of chains in which this concept occurs. Checking the list of chains against the SCOP classification gives the following results:

#Instances	SCOP classification	#Instances	SCOP classification
39	b.60.1.2	1	d.129.3.8
5	e.7.1.1	1	d.129.3.5
3	h.1.2.1	1	b.97.1.1
3	b.8.1.1	1	b.82.1.23
3	b.30.5.4	1	b.82.1.11
2	d.85.1.1	1	b.61.7.1
2	d.25.1.1	1	b.61.1.1
2	b.60.1.1	1	b.60.1.8
1	h.1.32.1	1	b.60.1.7
1	g.12.1.1	1	b.23.1.1
1	f.1.1.1	1	b.1.9.2
1	d.169.1.8	1	b.163.1.1

It is no surprise that most of these are from SCOP all-β class, as they are assemblies of β-strands, with class b.60.1.2, 'Fatty acid binding protein-like' as the exception. (However, because the *usage* option gives results for the entire wwPDB, the frequency may well reveal the experimental bias of solved structures within that family.)

Fig. SF2b shows the structural superposition of the regions from the linker region of the multimeric erythrocruorin from the earthworm, *Lumbricus terrestris,* (2GTL, chain O) and Human cellular retinol binding protein III (1GGL, chain A).

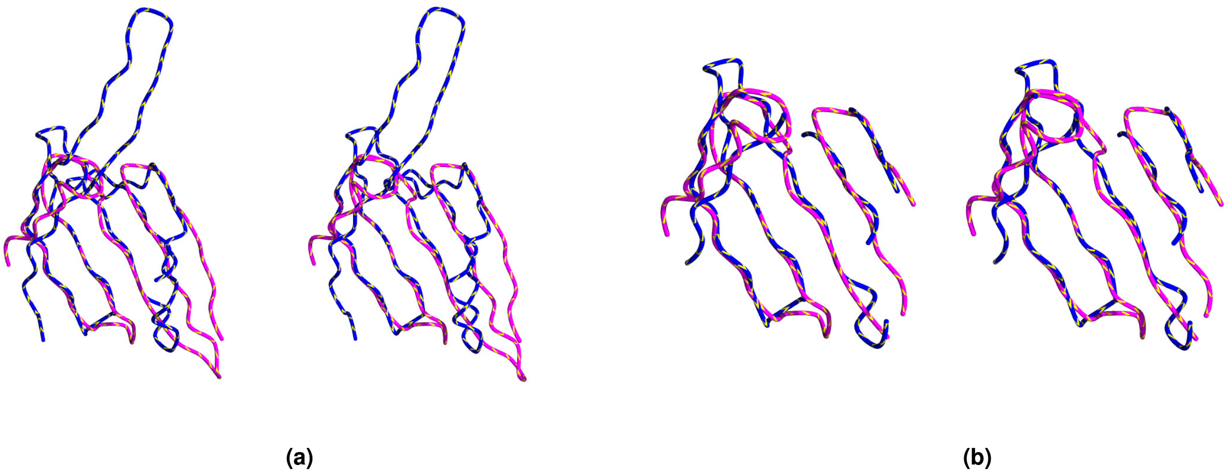

Fig. SF2. Regions of unrelated protein folds can share the same concepts.
Superposition (in stereo) of region containing concept from (blue) linker region of earthworm multimeric erythrocruorin and (pink) Human cellular retinol binding protein III. (a) Entire region comprising concept c_0559. (b) Restriction to well-fitting residues from region comprising concept c_0559.

Are the erythrocruorin linker domains and the retinol-binding proteins homologues? Structural comparison shows that only the regions of these proteins share secondary structural elements and the geometry of their assembly (as shown in Fig. SF2b). The rest of the domains are quite different. The regions are not homologues. However, the dictionary has identified a substructure which they, and other domains, share. Like other supersecondary structures, their appearance in unrelated proteins show that they are shared pieces of protein folds but not signs of homology. Indeed, an appeal to SCOP shows that the erythrocruorin linker domains are in a superfamily of their own, sharing a folding topology with 'Streptavidin-like' domains.

In summary, PROÇODIC provides useful information to study protein structures and identify shared substructural similarities between proteins, thus opening up to further explorations of their evolutionary antecedents.

References

1. Dietmann S, et al. (2001) A fully automatic evolutionary classification of protein folds: Dali Domain Dictionary version 3. *Nucleic acids research* 29(1):55–57.
2. Orengo C, Jones DT, Thornton JM (1994) Protein superfamilies and domain superfolds. *Nature* 372(6507):631.
3. Subramanian R, et al. (2017) Statistical compression of protein folding patterns for inference of recurrent substructural themes in *Data Compression Conference (DCC), 2017.* (IEEE), pp. 340–349.
4. Holm L, Sander C (1993) Structural alignment of globins, phycocyanins and colicin A. *FEBS Letters* 315(3):301–306.
5. Ruggiero Bachega JF, et al. (2015) The structure of the giant haemoglobin from *Glossoscolex paulistus*. *Acta Crystallographica. Section D, Biological Crystallography* 71(6):1257–1271.
6. Sievers F, Higgins DG (2014) Clustal Omega, accurate alignment of very large numbers of sequences. *Multiple Sequence Alignment Methods* pp. 105–116.
7. Blackshields G, Sievers F, Shi W, Wilm A, Higgins DG (2010) Sequence embedding for fast construction of guide trees for multiple sequence alignment. *Algorithms for Molecular Biology* 5(1):21.

Supplementary Figures

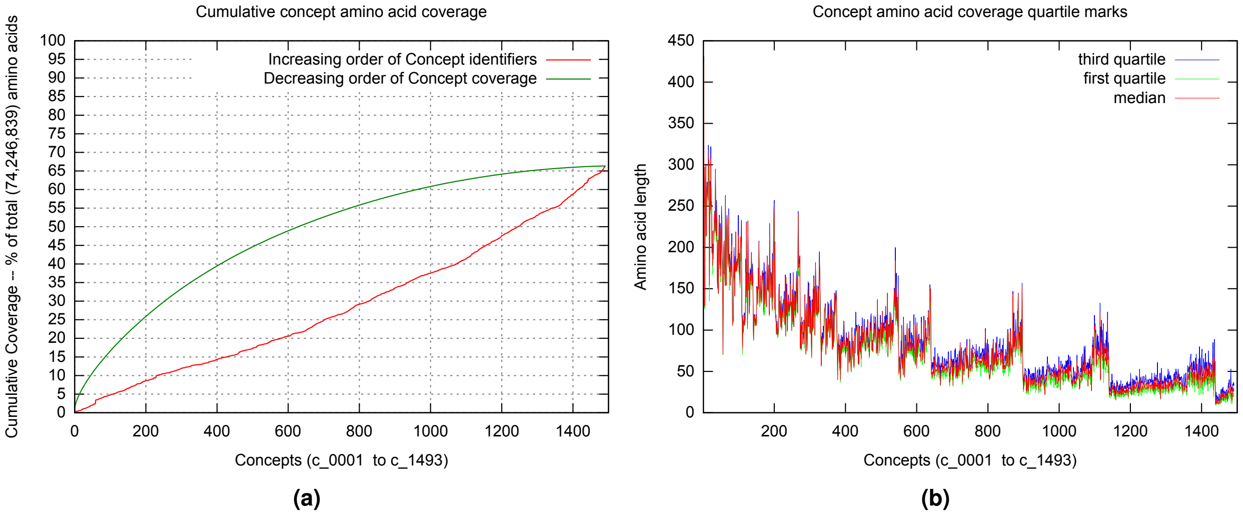

Fig. SF3. Cumulative and individual distributions of concepts' amino acid coverage.
(a) Cumulative amino acid coverage of concepts (as a percentage of the total 74, 246,839 number of residues) after dissecting 275,014 protein chains using the inferred dictionary. The green curve gives the distribution in the decreasing order of individual concept amino acid coverage – i.e., the concept with largest coverage is listed first, that with second largest coverage is listed second, and so on. The red curve gives the same cumulative distribution in the serial order of concept identifiers – i.e., concept `c_0001` is shown first, concept `c_0002` second and so on. (b) Dissecting the protein chains from the `wwPDB` allows us to catalogue the regions where each concept is used. Underlying each concept usage is an amino acid sequence of variable length (although the associated strings corresponding to the types and order of secondary structural elements match exactly). This graph plots the first, second and third quartile points in the distribution of amino acid lengths for each concept's set of usages in the `wwPDB`. Concepts are listed in the decreasing order of lengths, followed by the lexicographic order of their secondary structural strings. Since the average strand of sheet (denoted as 'E') has fewer amino acids than the average helix (denoted as 'H'), the lexicographic order creates in the plot the observed piecewise increasing trend among concepts with same number of secondary structural elements.

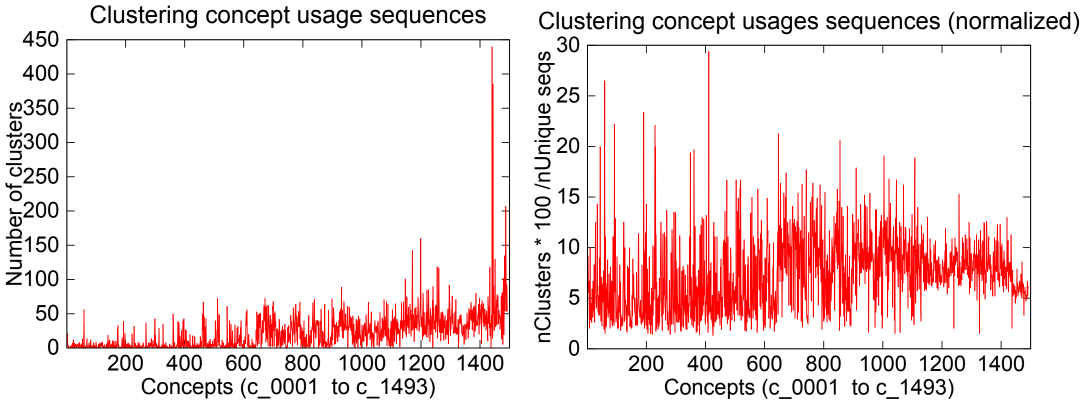

(a) (b)

Fig. SF4. Sequence clusters of concepts' usage-amino-acid-sequences.
(a) Number of clusters produced by `Clustal-Omega` (6) based on its computation of the multiple sequence alignment of the usage-amino-acids-sequences for each concept. `Clustal-Omega` uses the `mBed` algorithm to cluster sequences (7). (b) Normalised plot to account for the differences in the number of usages per concept. Normalization involves dividing the number of clusters by the total number of *unique* amino acid sequences observed in the set of usages per concept.
